# Assessment of Exposure to Essential and Non-Essential Elements through Concentrations in Tissues of *Chelonia mydas* (Green turtles) from the South Atlantic, Northeast Brazil

**DOI:** 10.1007/s12011-026-05303-2

**Published:** 2026-08-19

**Authors:** Midiã S. Rodrigues, Iago J. S. da Silva, Cristina F. da Fonseca, Pierre C. Soares, Múcio L. B. Fernandes, Geraldo J. B. de Moura

**Affiliations:** 1 Programa de Pós-Graduação Em Ciência Animal Tropical (PGCAT), Universidade Federal Rural de Pernambuco (UFRPE), Rua Dom Manoel de Medeiros, s/n, Dois Irmãos, Recife, Pernambuco 52171-9000, Brazil; 2 Laboratório de Estudos Herpetológicos E Paleherpertológicos (LEHP), Universidade Federal Rural de Pernambuco (UFRPE), Rua Dom Manoel de Medeiros, s/n, Dois Irmãos, Recife, Pernambuco 52171-9000 Brazil; 3 Unidade Acadêmica de Belo Jardim (UABJ), Universidade Federal Rural de Pernambuco (UFRPE), Rodovia PE 166, km 5, Euno Andrade da Silva, Belo Jardim, Pernambuco 55156-580 Brazil; 4 Laboratório de Parasitologia (LAPAR), Departamento de Biologia, Universidade Federal Rural de Pernambuco (UFRPE), Rua Dom Manoel de Medeiros, s/n, Dois Irmãos, Recife, Pernambuco 52171-9000 Brazil; 5 Departamento de Medicina Veterinária, Universidade Federal Rural de Pernambuco (UFRPE), Rua Dom Manoel de Medeiros, s/n, Dois Irmãos, Recife, Pernambuco 52171-9000 Brazil; 6 Laboratório de Estudos Ambientais, Universidade de Pernambuco (UPE), Rua Amaro Maltês de Farias, Nazaré da Mata, Pernambuco 55800-000 Brazil; 7 Programa de Pós-Graduação Em Ecologia (PPGE), Universidade Federal Rural de Pernambuco (UFRPE), Rua Dom Manoel de Medeiros, s/n, Dois Irmãos, Recife, Pernambuco 52171-9000 Brazil

**Keywords:** Metals, Sea turtles, Foraging, Toxicity, Food web, Bioaccumulation

## Abstract

This study aimed to evaluate the exposure of *Chelonia mydas* (green turtles) inhabiting the south coast of Pernambuco, Northeast Brazil, to essential and toxic elements. Concentrations of Al, Cd, Co, Cr, Cu, Fe, Mn, Mo, Ni, Pb, and Zn were determined in shell, muscle, liver, and kidney samples from 43 individuals using ICP-OES. Element concentrations varied according to tissue type. Specifically, the average concentrations of Al in shell (1064 mg kg^−1^ d.w.) and Fe in liver (2069 mg kg^−1^ d.w.) were the highest, whereas Al, Co, Cr, and Mo in muscle were the lowest and fell below the method's limit of quantification (LOQ). Meanwhile, Pb was above the LOQ only in muscle (average of 4.1 mg kg^−1^ d.w.). Cadmium levels were markedly elevated, with average concentrations of 0.15, 0.29, 12, and 49 mg kg^−1^ d.w. in shell, muscle, liver, and kidney, respectively; furthermore, concentrations in muscle, liver, and kidney were well above the limits established by Brazilian legislation (0.05, 0.5, and 1.0 mg kg^−1^, respectively). High levels of Cd may be linked to the presence of fertilizers in coastal areas, the ingestion of contaminated food, and chronic exposure. Notably, fibropapillomatosis (FP) showed a significant relationship with shell Cu content, demonstrating that this metal is linked to the etiology and development of FP. Overall, multi-elemental profiling of sea turtle tissues can serve as a cost-effective surveillance tool for marine ecosystem monitoring and for detecting anthropogenic impacts on sea turtle health.

**Supplementary Information:**

The online version contains supplementary material available at https://doi.org/10.1007/s12011-026-05303-2.

## Introduction

Marine ecosystems are exposed to contamination by a wide range of pollutants caused by anthropogenic activities such as agriculture and industry [1]. Chemical pollution in the marine environment is widespread due to the common use of chemicals and metals throughout the world, and the tendency for transport and persistence of many of these compounds [2–6].

Essential elements, such as Cu, Fe, Mn, Se, and Zn, play fundamental roles in biological systems, acting as structural components of biomolecules, enzyme cofactors, and regulators of metabolic and antioxidant processes. Although indispensable for normal physiological functions, these elements may also exert toxic effects when present at concentrations exceeding the organism's homeostatic capacity. In contrast, non-essential elements, including As, Cd, Hg, and Pb, have no known physiological function and may accumulate in tissues, causing adverse effects on the nervous, immune, reproductive, and endocrine systems, as well as oxidative stress and other toxic responses. Therefore, evaluating both essential and non-essential elements is crucial for assessing the nutritional status and contaminant exposure of wildlife populations [7, 8].

Although some of these essential elements are natural components of rocks and soil, present in the marine environment due to climate and erosion [9, 10], human activities are causing unnatural quantities of these elements to accumulate in these ecosystems, altering their natural biological balance [5, 6, 11–15].

Some of these contaminants can remain for an extended period in the marine environment, thus affecting the health of many organisms, leading to changes in the structure of the food web and influencing bioaccumulation in the tissues of plants and animals, including sea turtles [6, 16, 17]. These reptiles are potential bioaccumulators of polluting elements, since they are animals with late sexual maturity, long life, and migratory habits, commonly living in a variety of foraging habitats during their development [6, 18–21]. Sea turtles are hence greatly exposed to pollution caused by the discharge of waste into the marine environment [6, 12, 22].

Studies have identified organic and inorganic contaminants in the blood, tissue, and eggs of sea turtles around the world [1–3, 6, 23–34]. However, little is known about the toxicological effects of anthropogenic chemical contaminants on sea turtles or how they can affect the health of these animals [4, 5, 12–15, 18, 35].

The species *Chelonia mydas* in particular has characteristics that put it at risk of these pollutants, especially in Brazilian waters. *C. mydas* is the most coastal species among those that live in the South Atlantic offshore from Brazil [36]. It has high dietary plasticity, with ontogenetic changes in its eating habits. When reaching curvilinear carapace length (CCL) between 30 and 40 cm, it becomes preferentially herbivorous, feeding mainly on seagrasses and benthic algae [6, 14, 21, 37–40]. In polluted areas, toxic elements are typically higher in the surrounding waters and sediments, as well as in seagrass and algae [41].

In addition, studies have shown that diseases such as fibropapillomatosis (FP) are more frequent in green turtles in coastal, shallow, and polluted regions [42, 43], and evidence suggests that exposure to toxic metals may act synergistically in the etiology and development of FP [10, 23, 44–53]. To date, no multi-tissue and multi-element study has characterized the mineral profile of *C. mydas* on the southern coast of Pernambuco, leaving a critical data gap for regional biomonitoring programs.

Therefore, this work aimed to evaluate the concentrations and distribution of essential and non-essential elements (Al, Cd, Co, Cr, Cu, Fe, Mn, Mo, Ni, Pb, and Zn) in liver, kidney, muscle, and shell samples of *C. mydas* found along the south coast of Pernambuco, Northeast Brazil. This knowledge is important since it can be used to monitor changes in the bioavailability of these essential and non-essential elements in the habitat of these sea turtles, providing baseline data for future monitoring studies of this and other species and to determine whether these elements are present in the tissues in concentrations that can negatively impact the health of the population.

## Material and Methods

### Study Areas and Obtaining Samples

**Fig. 1:**
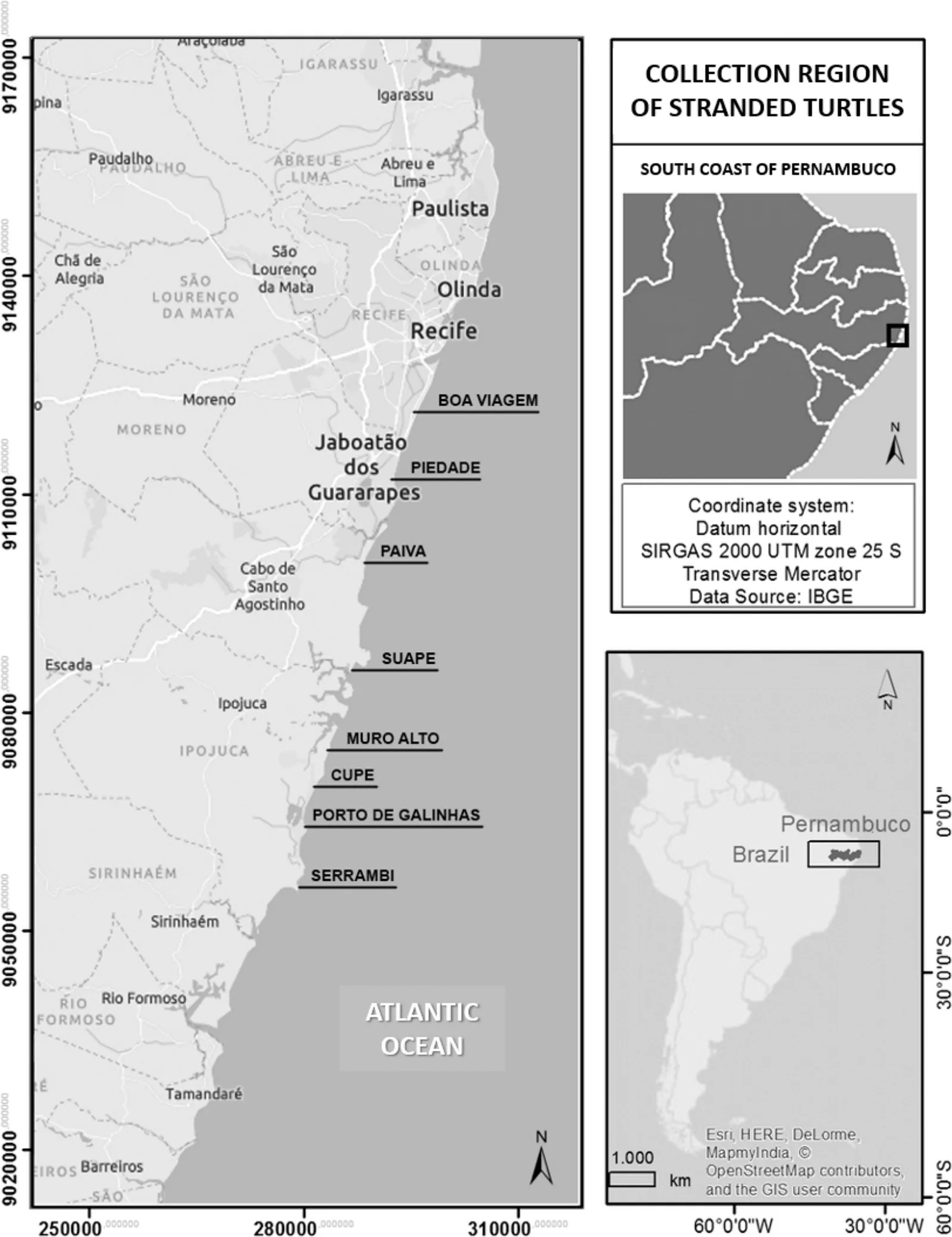
Sampling sites for *Chelonia mydas* populations along the South Coast of Pernambuco, Northeast Brazil. IBGE—Brazilian Institute of Geography and Statistics

The study sites corresponded to eight beaches on the south coast of the state of Pernambuco (−8.078341/−34.875720 and −8.606433/−35.046698), Northeastern Brazil, in the municipalities of Recife, Jaboatão dos Guararapes, Cabo de Santo Agostinho, and Ipojuca (Fig. 1). The study was done by dividing the investigation site into Areas 1, 2, and 3, which were composed of the following beaches: Area 1 (Boa Viagem and Piedade), Area 2 (Paiva and Suape), and Area 3 (Muro Alto, Cupe, Porto de Galinhas, and Serrambi). This work was carried out from November 2016 to November 2018, during which 43 stranded *C. mydas* specimens were collected and analyzed.

The fresh carcasses (carcass condition: code 2) were transported to the Herpetological and Paleoherpetological Studies Laboratory (LEHP) of Federal Rural University of Pernambuco (UFRPE) where they were necropsied. During that process, the sex of the animals was determined from visual analysis of the gonads [54, 55]. Biometric measurements were made with a tape measure, such as the curvilinear carapace length (CCL) and curvilinear carapace width (CCW), in order to ascertain the development stage of the turtles [56].

Whenever feasible, liver, pectoral muscle, kidney, and peripheral shell bone samples were collected for elemental determination. The samples were individually stored in clean containers without chemical preservatives and kept frozen at − 20 °C until processing. The number of samples analyzed for each tissue was as follows: liver (*n* = 39), pectoral muscle (*n* = 43), kidney (*n* = 38), and shell (*n* = 43).

This project was authorized under Biodiversity Authorization and Information System (Sisbio) license No. 56195–2, in compliance with Normative Instruction 154/2007 from the Brazilian Institute for the Environment and Renewable Natural Resources (IBAMA), which allows the collection and transport of animals found dead for scientific or didactic use by accredited scientific or teaching institutions.

### Preparation of Tissues

The muscle, liver, and kidney samples were thawed under refrigeration, placed in identified Petri dishes and dried in an oven with controlled temperature between 55 °C and 60 °C for approximately 24 h or until constant mass was achieved. Subsequently, the material was homogenized using a mortar and pestle to obtain a fine homogeneous powder. To avoid cross contamination, the tissues were dried in the oven separately, grouped by type.

### Assessment of Digestion Efficiency

Before defining the digestion method, tests were performed with one sample of each type of tissue to assess the efficiency of the decomposition procedure. This assessment was carried out by determining the residual carbon content (RCC). Adjustments were made to the digestion method (sample mass, digestion time, and temperature) until acceptable RCC values for biological materials (up to 35%) were obtained.

The RCC is defined as the percentage (by mass) of carbon remaining in the sample after the decomposition process, in relation to the original carbon content (OCC) in the sample before digestion [57]. The quantification of the OCC and RCC was performed with a UV–Visible molecular absorption spectrophotometer (DR/2010, Hach, USA). This determination was carried out following the procedure proposed by a Silva et al. [57, 58], which is briefly described below.

First, a 1 mL aliquot (solution or suspension) or 6 mg of sample (solid) was transferred directly to threaded test tubes (Hach, USA). Then 1.5 mL of K_2_Cr_2_O_7_ (50 g L^−1^) and 2.5 mL of 96% H_2_SO_4_ (Química Moderna, Brazil) were added. The tubes were closed, placed in a vortex shaker (Q-220B2, Quimis, Brazil) to homogenize the contents and then placed in a block digester (Spectroquant TR 420, Merck, Germany) at 150 °C for 2 h. Then, after cooling to room temperature, the absorbance was measured at a 620 nm wavelength using the tube itself as cuvette (13.5 mm path length). The carbon concentration was estimated using a previously plotted analytical curve. When the carbon content was very high, the sample was diluted (solution or suspension) or used in masses below 6 mg (solid).

A stock solution of potassium hydrogen phthalate (Merck, Germany) containing 10,000 mg L^−1^ of carbon was prepared, from which standard calibration solutions were prepared in the range of 50—3200 mg L^−1^ of carbon.

### Microwave-Assisted Acid Digestion

The samples were prepared before quantifying the minerals by acid digestion assisted by microwave radiation. The analytical blank solutions were processed in triplicate and went through all the sample preparation steps described in detail below, with the absence of samples.

#### Muscle

The digestion of muscle samples was carried out using a Multiwave 3000 microwave oven from Anton Paar (Austria) with perfluoroalkoxy (PFA) digestion flasks. For muscle samples, a mass of approximately 500 mg was measured on an analytical balance and transferred to the digestion flasks. Then, 5 mL of 65% HNO_3_ was added, the flasks were closed, and the following heating program was conducted: (a) 10 min to reach 100 ºC; (b) 2 min hold at 100 ºC; (c) 5 min to reach 140 ºC; (d) 20 min hold at 140 ºC; and 30 min for cooling the digestion flasks. The digested samples were transferred to conical polypropylene tubes and the volume was completed to 15 mL with distilled-deionized water.

#### Liver and Kidney

The digestion of liver and kidney samples was carried out in a microwave oven (DGT 100 Plus, Provecto Analítica, Brazil) with modified polytetrafluoroethylene (TFM) digestion flasks. For liver samples, a mass of approximately 500 mg was measured and transferred to the digestion flasks, followed by the addition of 5 mL of 65% HNO_3_. In the case of kidney samples, 300 mg or the mass that was obtained after the drying step was measured and transferred to the flasks. Then, 5 mL of 65% HNO_3_ was added, the flasks were closed, and the following heating program was applied: (a) 330 W for 5 min; (b) 660 W for 5 min; (c) 900 W for 4 min; and (d) 0 W for 7 min. The digested samples were transferred to conical polypropylene tubes and the volume was completed to 15 mL with distilled-deionized water. The microwave oven employed operates with a heating program adjusted by using microwave radiation power instead of temperature. Each digestion round was carried out using six flasks.

#### Peripheral Bone Shell

The digestion of sea turtle shell samples was carried out in a microwave oven (DGT 100 Plus, Provecto Analítica, Brazil) with TFM digestion flasks. For shell samples, a mass of approximately 200 mg was measured and transferred to the digestion flasks, followed by the addition of 5 mL of 65% HNO_3_ plus 2.5 mL of 96% H_2_SO_4_. The flasks were closed and the following heating program was applied: (a) 330 W for 5 min; (b) 600 W for 5 min; (c) 800 W for 3 min; and (d) 0 W for 6 min. The digested samples were transferred to conical polypropylene tubes, and the volume was completed to 15 mL with distilled-deionized water. The microwave oven employed operates with a heating program adjusted by using microwave radiation power instead of temperature. Each digestion round was carried out using six flasks.

### Determination of the Limits of Quantification (LOQ)

To determine the LOQ of the method, 10 analytical blanks were prepared following the digestion procedure established for each type of sample (peripheral bone shell, muscle, liver, and kidney). The LOQ is defined as 10 × SD_blank_/s, where “SD_blank_” is the standard deviation of the analytical signal of the blanks (*n* = 10) and “s” is the slope of the analytical curve [59, 60]. Only concentration values that were above the LOQ were expressed and discussed in this work.

### Mineral Determination

The concentrations of Al, Cd, Co, Cr, Cu, Fe, Mn, Mo, Ni, Pb, and Zn were determined using an inductively coupled plasma optical emission spectrometer (ICP-OES) (Model 5100, Agilent Technologies, USA). The following operating conditions were used: radiofrequency power of 1200 W, plasma gas flow rate of 12 L min^−1^, auxiliary gas flow rate of 1.0 L min^−1^, nebulization gas flow rate of 1.0 L min^−1^, sample flow rate of 1.0 mL min^−1^, flush time of 10 s, delay time of 15 s, a concentric nebulizer, and a cyclonic spray chamber.

The following emission lines (nm) were monitored in the axial view of the plasma: Al I 396.152, Cd II 214.439, Co II 228.615, Cr II 267.716, Cu I 327.395, Fe II 238.204, Mn II 257.610, Mo II 202.032, Ni II 231.604, Pb II 220.353, and Zn I 213.857. Argon with minimum purity of 99.999% (Messer Gases, Brazil) was used in ICP-OES for plasma generation, nebulization, and as auxiliary gas.

The multi-elemental analytical curve was prepared from mono-elemental solutions of the analytes Al, Cd, Cr, Cu, Fe, Mn, Pb, Zn (Specsol, Brazil), Co, Mo, (SCP Science, Canada) and Ni (Inorganic Ventures, USA), at a concentration of 1000 mg L^−1^. The following concentrations of the standard calibration solution were applied in the preparation of the analytical curve: 0.5, 10, 20, 30, 40, and 50 mg L^−1^ (Al, Fe); 0.05, 1, 2, 3, 4, and 5 mg L^−1^ (Cu, Mn, Zn); 0.005, 0.1, 0.2, 0.3, 0.4, and 0.5 mg L^−1^ (Cr, Ni, Pb); and 0.001, 0.02, 0.04, 0.06, 0.08, and 0.1 mg L^−1^ (Cd, Co, Mo). The solutions were prepared in 5% (v v^−1^) HNO_3_ medium to increase their stability to avoid the precipitation and/or adsorption of the analytes in the containers.

### Statistical Analysis

The means and standard deviations of the data obtained were calculated. The parameters were tested initially for normal distribution, using the Kolmogorov–Smirnov test. Those which did not meet the assumptions of normality and homogeneity of variance were submitted to logarithmic transformation (Log (X + 1)) or square root transformation (√(x + 1)). Subsequently, the data were subjected to analysis of variance (ANOVA, F-test), partitioned the following sources of variation: collection area (area 1, *N* = 17; area 2, *N* = 4; area 3, *N* = 22), sex (male, *N* = 7; female, *N* = 36), and FP status (presence, *N* = 11; absence, *N* = 32). A limitation of the analysis's statistical power is the small sample size of individuals in area 2 (*N* = 4) and of males (*N* = 7). When the F-test indicated a significant effect, pairwise comparisons among least-squares means were performed using the LSMEANS statement with a Bonferroni adjustment to control the Type I error associated with multiple pairwise comparisons. Statistical analyses were performed using the General Linear Model (GLM) procedure of the Statistical Analysis System (SAS, version 9.1; SAS Institute Inc., USA). Statistical significance was set at *p* < 0.05.

Also, an exploratory study was performed applying principal component analysis (PCA) to the mineral composition data. All computations were carried out using The Unscrambler X software (Version 10.4, CAMO, Norway). The data matrix was composed of 43 samples and 11 variables (Al, Cd, Co, Cr, Cu, Fe, Mn, Mo, Ni, Pb, and Zn). All data were auto scaled before processing and a full cross validation method was performed to validate the model. Both ANOVA and PCA were used to evaluate the effect of collection area, sex, and FP on the mineral profile of the analyzed tissues.

## Results

Forty-three dead stranded turtles were collected along the southern coast of Pernambuco and analyzed. Among these, 11 had FP. The average carapace size was 46.58 ± 14.82 cm, varying between 32 and 111 cm. Most animals (32 individuals) had carapace length between 32 and 49 cm, the rest were between 50 and 111 cm. Among the 43 animals found, 7 were males and 36 females. Juveniles were considered to be *C. mydas* individuals with CCL up to 95 cm, based on the minimum size of breeding females on Brazilian beaches [61]. Therefore, only one individual was classified as an adult, limiting the interpretation of patterns related to body size, age, and population structure. Taking into account the different stranding areas, 17, 4, and 22 individuals were collected in Areas 1, 2, and 3, respectively.

### Evaluation of Digestion Efficiency and LOQ

A sample of each material (peripheral bone shell, liver, muscle, and kidney) was selected at random for determination of the RCC. The following values were obtained: 34.9 ± 0.1% for shell, 22 ± 2% for liver, 21.9 ± 0.5% for muscle, and 20.4 ± 0.6% for kidney. The OCC values were also quantified, and the following results were achieved: 37 ± 3% for peripheral bone shell, 46 ± 1% for liver, 43 ± 3% for muscle, and 47 ± 2% for kidney. The LOQ values of the method for each element determined are shown in Table 1.

**Table 1:** Limits of quantification of the method for the different elements and types of biological samples of *Chelonia mydas* evaluated

Samples (mg kg^−1^)	Al	Cd	Co	Cr	Cu	Fe	Mn	Mo	Ni	Pb	Zn
Shell	7.9	0.14	0.33	0.20	0.47	2.3	0.042	0.61	2.2	5.1	11
Liver	4.0	0.050	0.19	0.11	0.35	3.3	0.055	0.28	2.2	6.1	12
Muscle	1.7	0.053	0.18	0.13	0.21	3.1	0.099	0.28	0.46	1.4	1.7
Kidney	4.0	0.050	0.19	0.11	0.35	3.3	0.055	0.28	2.2	6.1	12

### Mineral Profile of Biological Samples of *C. mydas*

Table 2 presents the mineral profile of the samples, specified by tissue type. Tables 3, 4, 5 and 6 show the multi-elemental concentrations of the shell (Table 3), liver (Table 4), muscle (Table 5), and kidney (Table 6) samples, considering the sex, the presence of FP, and the stranding area of the animals. The values below the LOQ are indicated with asterisks in Tables 2, 3, 4, 5 and 6.

**Table 2:** Composition of essential and toxic elements in biological samples of *Chelonia mydas* along the southern coast of Pernambuco, Northeast Brazil

Samples	Elements in mg kg^−1^, mean value (concentration range)										
	Al	Cd	Co	Cr	Cu	Fe	Mn	Mo	Ni	Pb	Zn
Shell	1064 (148–1865)	0.15 (< 0.14–0.19)	0.44 (< 0.33–0.81)	44 (5.8–150)	2.7 (0.59–7.8)	289 (57–1243)	17 (1.5–172)	1.5 (< 0.61–2.8)	13 (< 2.2–27)	*	258 (83–422)
Liver	441 (< 4.0–928)	12 (3.5–44)	0.78 (0.29–3.1)	15 (< 0.11–32)	65 (6.4–212)	2069 (315–11591)	7.0 (0.42–17)	0.89 (< 0.28–3.1)	4.4 (< 2.2–11)	*	82 (31–193)
Muscle	*	0.29 (< 0.053–4.6)	*	*	1.5 (< 0.21–35)	28 (< 3.1–948)	0.36 (< 0.099–5.6)	*	16 (< 0.46–31)	4.1 (< 1.4–7.4)	74 (18–225)
Kidney	644 (248–808)	49 (8.7–177)	3.2 (0.60–10)	22 (7.5–30)	8.4 (3.2–38)	494 (147–939)	5.6 (2.9–12)	0.89 (< 0.28–2.3)	5.9 (< 2.2–8.1)	*	87 (43–189)

^*^Values below the LOQ of the method

**Table 3:** Composition of essential and toxic elements in **shell of *Chelonia mydas*** along the southern coast of Pernambuco, Northeast Brazil, considering the sex, the presence of fibropapillomatosis, and the stranding area of the animals

Evaluated factors	Elements in mg kg^−1^, mean value (concentration range)^a^										
	Al	Cd	Co	Cr	Cu	Fe	Mn	Mo	Ni	Pb	Zn
Sex											
Male	1407 (321–1780)	0.15 (< 0.14–0.18)	0.49 (< 0.33–0.71)	53 (35–62)	3.1 (1.7–4.2)	333 (294–372)	13 (6.6–41)	1.8 (0.66–2.2)	18 (2.3–24)	*	281 (213–386)
Female	997 (148–1865)	0.15 (< 0.14–0.19)	0.44 (< 0.33–0.81)	42 (5.8–150)	2.6 (0.59–7.8)	280 (57–1243)	18 (1.5–172)	1.5 (< 0.61–2.8)	12 (< 2.2–27)	*	254 (83–422)
Fibropapillomatosis											
Presence	1283 (399–1865)	0.14 (< 0.14–0.17)	0.49 (< 0.33–0.81)	62 (15–150)	**3.3 A** (0.92–4.5)	427 (102–1243)	37 (2.7–172)	1.8 (< 0.61–2.8)	17 (3.2–27)	*	259 (83–422)
Absence	989 (148–1780)	0.15 (< 0.14–0.19)	0.43 (< 0.33–0.71)	38 (5.8–74)	**2.5 B** (0.59–7.8)	241 (57–511)	11 (1.5–51)	1.4 (< 0.61–2.8)	12 (< 2.2–24)	*	258 (112–393)
Stranding area											
Area 1	1160 (264–1737)	0.15 (< 0.14–0.18)	0.47 (< 0.33–0.70)	49 (9.4–84)	3.5 (0.62–7.8)	323 (72–760)	19 (2.7–145)	1.7 (< 0.61–2.8)	15 (< 2.2–24)	*	290 (154–422)
Area 2	775 (344–1780)	0.16 (< 0.14–0.19)	0.43 (< 0.33–0.71)	28 (13–62)	1.9 (0.92–4.2)	178 (93–372)	4.7 (2.9–8.2)	1.2 (< 0.61–2.2)	8.7 (< 2.2–24)	*	276 (83–393)
Area 3	1042 (148–1865)	0.14 (< 0.14–0.19)	0.43 (< 0.33–0.81)	43 (5.8–150)	2.3 (0.59–4.5)	282 (57–1243)	18 (1.5–172)	1.4 (< 0.61–2.8)	12 (< 2.2–27)	*	231 (112–374)

^*^Values below the LOQ of the method

^a^Mean values followed by different letters and highlighted in **bold** show a statistically significant difference at a 95% confidence level

**Table 4:** Composition of essential and toxic elements in **liver of *Chelonia mydas*** along the southern coast of Pernambuco, Northeast Brazil, considering the sex, the presence of fibropapillomatosis, and the stranding area of the animals

Evaluated factors	Elements in mg kg^−1^, mean value (concentration range)										
	Al	Cd	Co	Cr	Cu	Fe	Mn	Mo	Ni	Pb	Zn
Sex											
Male	371 (< 4.0–817)	9.7 (5.4–15)	0.59 (0.50–0.91)	12 (< 0.11–29)	70 (15–171)	1626 (971–2630)	6.9 (2.1–14)	0.73 (< 0.28–1.5)	4.2 (< 2.2–8.6)	*	81 (31–115)
Female	456 (< 4.0–928)	13 (3.5–44)	0.82 (0.29–3.1)	15 (< 0.11–32)	63 (6.4–212)	2166 (315–11,591)	7.1 (0.42–17)	0.93 (< 0.28–3.1)	4.5 (< 2.2–11)	*	82 (33–193)
Fibropapillomatosis											
Presence	297 (< 4.0–675)	12 (5.4–38)	0.68 (0.34–1.5)	9.2 (< 0.11–21)	47 (9.7–106)	2426 (826–5359)	7.7 (1.5–17)	0.92 (< 0.28–2.3)	3.3 (< 2.2–6.0)	*	91 (31–193)
Absence	490 (< 4.0–928)	12 (3.5–44)	0.81 (0.29–3.1)	16 (< 0.11–32)	71 (6.4–212)	1946 (315–11,591)	6.8 (0.42–14)	0.88 (< 0.28–3.1)	4.8 (< 2.2–11)	*	79 (33–127)
Stranding area											
Area 1	419 (< 4.0–746)	12 (6.0–38)	0.76 (0.34–1.5)	13 (< 0.11–21)	62 (21–120)	2734 (541–11,591)	7.8 (1.5–17)	1.0 (< 0.28–3.1)	3.6 (< 2.2–5.3)	*	98 (47–193)
Area 2	429 (< 4.0–675)	24 (12–44)	1.7 (0.91–3.1)	14 (< 0.11–21)	138 (30–212)	1832 (1175–2529)	10 (6.1–14)	1.2 (< 0.28–2.3)	4.1 (< 2.2–5.2)	*	87 (37–115)
Area 3	456 (< 4.0–928)	11 (3.5–28)	0.67 (0.29–1.8)	15 (< 0.11–32)	56 (6.4–177)	1679 (315–5958)	6.1 (0.42–14)	0.77 (< 0.28–1.7)	4.9 (< 2.2–11)	*	71 (31–127)

^*^Values below the LOQ of the method

**Table 5:** Composition of essential and toxic elements in **muscle of *Chelonia mydas*** along the southern coast of Pernambuco, Northeast Brazil, considering the sex, the presence of fibropapillomatosis, and the stranding area of the animals

Evaluated factors	Elements in mg kg^−1^, mean value (concentration range)										
	Al	Cd	Co	Cr	Cu	Fe	Mn	Mo	Ni	Pb	Zn
Sex											
Male	*	0.18 (0.12–0.30)	*	*	0.61 (< 0.21–1.3)	8.1 (< 3.1–24)	*	*	17 (< 0.46–29)	4.4 (< 1.4–6.9)	74 (27–116)
Female	*	0.32 (< 0.053–4.6)	*	*	1.7 (< 0.21–35)	32 (< 3.1–948)	0.41 (< 0.099–5.6)	*	16 (< 0.46–31)	4.1 (< 1.4–7.4)	74 (18–225)
Fibropapillomatosis											
Presence	*	0.23 (< 0.053–0.52)	*	*	0.89 (0.25–1.5)	7.1 (< 3.1–26)	0.56 (< 0.099–4.4)	*	14 (< 0.46–30)	3.7 (< 1.4–7.4)	76 (29–145)
Absence	*	0.32 (< 0.053–4.6)	*	*	1.7 (< 0.21–35)	35 (< 3.1–948)	0.28 (< 0.099–5.6)	*	17 (< 0.46–31)	4.3 (< 1.4–7.4)	73 (18–225)
Stranding area											
Area 1	*	0.20 (< 0.053–0.62)	*	*	0.80 (< 0.21–2.2)	7.4 (< 3.1–71)	0.39 (< 0.099–4.4)	*	17 (< 0.46–31)	4.2 (< 1.4–7.4)	77 (27–145)
Area 2	*	0.24 (0.065–0.52)	*	*	0.72 (< 0.21–1.3)	*	*	*	7.1 (< 0.46–27)	2.7 (< 1.4–6.6)	57 (18–116)
Area 3	*	0.37 (< 0.053–4.6)	*	*	2.2 (< 0.21–35)	48 (< 3.1–948)	0.35 (< 0.099–5.6)	*	17 (< 0.46–31)	4.3 (< 1.4–7.4)	75 (19–225)

^*^Values below the LOQ of the method

**Table 6:** Composition of essential and toxic elements in **kidney of *Chelonia mydas*** along the southern coast of Pernambuco, Northeast Brazil, considering the sex, the presence of fibropapillomatosis, and the stranding area of the animals

Evaluated factors	Elements in mg kg^−1^, mean value (concentration range)^a^										
	Al	Cd	Co	Cr	Cu	Fe	Mn	Mo	Ni	Pb	Zn
Sex											
Male	652 (551–772)	43 (18–67)	**5.1 A** (1.5–10)	22 (18–27)	6.6 (4.7–10)	570 (370–725)	5.3 (3.6–8.9)	0.87 (0.71–1.0)	6.2 (4.4–8.1)	*	81 (51–99)
Female	643 (248–808)	51 (8.7–177)	**2.8 B** (0.60–7.6)	22 (7.5–30)	8.8 (3.2–38)	477 (147–939)	5.6 (2.9–12)	0.89 (< 0.28–2.3)	5.8 (< 2.2–8.1)	*	88 (43–189)
Fibropapillomatosis											
Presence	598 (248–751)	33 (8.7–62)	3.2 (0.60–9.7)	20 (7.5–30)	9.8 (3.2–38)	405 (147–822)	5.9 (3.7–8.9)	0.93 (< 0.28–2.3)	5.1 (< 2.2–8.0)	*	81 (43–189)
Absence	659 (534–808)	54 (18–177)	3.2 (0.73–10)	23 (18–28)	7.9 (4.7–24)	522 (194–939)	5.5 (2.9–12)	0.88 (0.62–1.2)	6.1 (3.6–8.1)	*	88 (51–177)
Stranding area											
Area 1	597 (248–779)	49 (18–86)	2.9 (0.60–7.6)	20 (7.5–30)	10 (3.4–38)	471 (147–939)	6.0 (3.0–12)	0.94 (< 0.28–2.3)	5.1 (< 2.2–8.1)	*	94 (43–189)
Area 2	708 (582–805)	52 (25–90)	5.8 (3.6–10)	24 (21–26)	13 (5.0–24)	481 (213–683)	5.8 (3.6–8.1)	0.89 (0.71–0.99)	6.7 (4.4–8.0)	*	74 (61–99)
Area 3	659 (496–808)	49 (8.7–177)	2.9 (0.73–9.7)	23 (13–28)	6.4 (3.2–15)	509 (256–757)	5.3 (2.9–8.9)	0.86 (0.49–1.2)	6.1 (< 2.2–8.1)	*	85 (51–177)

^*^Values below the LOQ of the method

^a^Mean values followed by different letters and highlighted in **bold** show a statistically significant difference at a 95% confidence level

Most elements were present in quantifiable concentrations in almost all samples (Table 2), with the exception of Pb, which was above the limit of quantification only in muscle. The organotropism of some elements was clear. Aluminum, Cr, and Fe concentrations were higher in shell, kidney, and liver, while Cd had higher values in kidney and liver. For Co, the highest concentration was found in kidney, with low concentrations in other tissues; Mn showed low concentrations in muscle, but in shell, liver and kidney, the concentrations were higher. For Ni, the highest concentrations were in muscle and shell, followed by kidney and liver. The element Zn was present at high concentrations in the shell but showed a pattern of lower concentrations in the liver, muscle, and kidneys. The highest concentration of Cu was found in the liver, while the highest concentration of Mo was in the shell. Muscle tissue had the lowest concentrations of all elements, except for Cd, Ni, and Pb. One alarming finding that needs to be highlighted was the high levels of Cd in the liver and kidney, with concentration ranges of 3.5–44 mg kg^−1^ and 8.7–177 mg kg^−1^, respectively (Table 2). For most elements, particularly Cd and Co, the pattern of accumulation in the tissues, in general, followed the order: kidney > liver > shell > muscle.

The results of the parametric ANOVA and the Student–Newman–Keuls post-hoc tests did not indicate significant differences (p > 0.05) relating the determined elements and the biometric measurements (CCL, CCW, and weight), in any of the variation factors (shell, liver, muscle, and kidneys).

### Effect of the Sex, Fibropapillomatosis, and Stranding Area on the Mineral Accumulation in Biological Samples

Considering the 11 elements determined across four tissue types (carapace, liver, muscle, and kidney), a total of 44 comparisons were performed for each explanatory factor (sex, fibropapillomatosis status, and stranding area). Among these comparisons, only two statistically significant differences were observed. The Cu concentrations were higher in the carapace of turtles with fibropapillomatosis than in those without the disease (*p* = 0.0246; Table 3), whereas Co concentrations were higher in the kidneys of males than in females (*p* = 0.0205; Table 6).

**Fig. 2:**
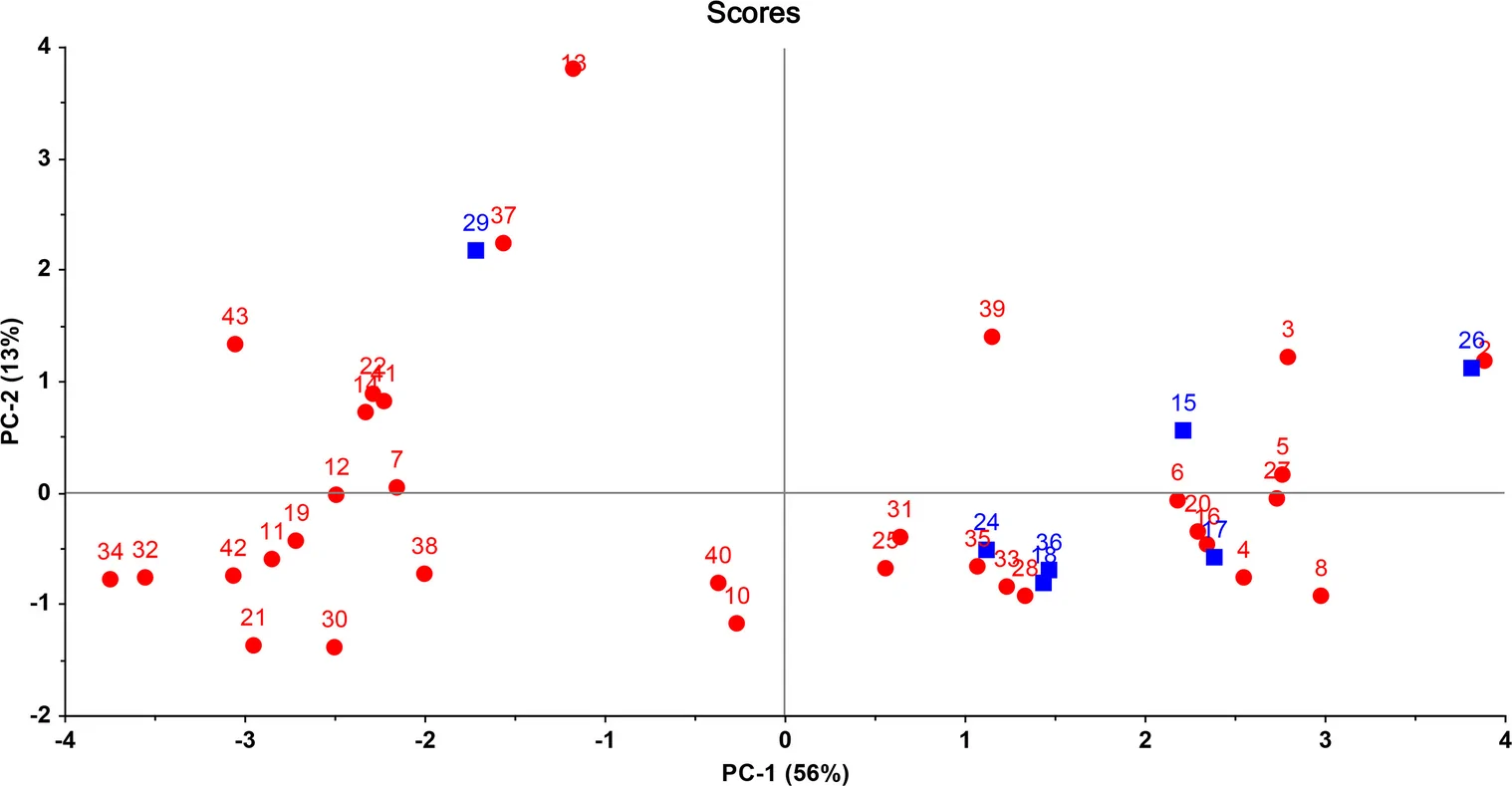
Scores plot of PCA for shell samples specified by sex: male (blue boxes) and female (red dots). PC—principal component. (For interpretation of the references to color in this figure legend, the reader is referred to the web version of this article)

**Fig. 3:**
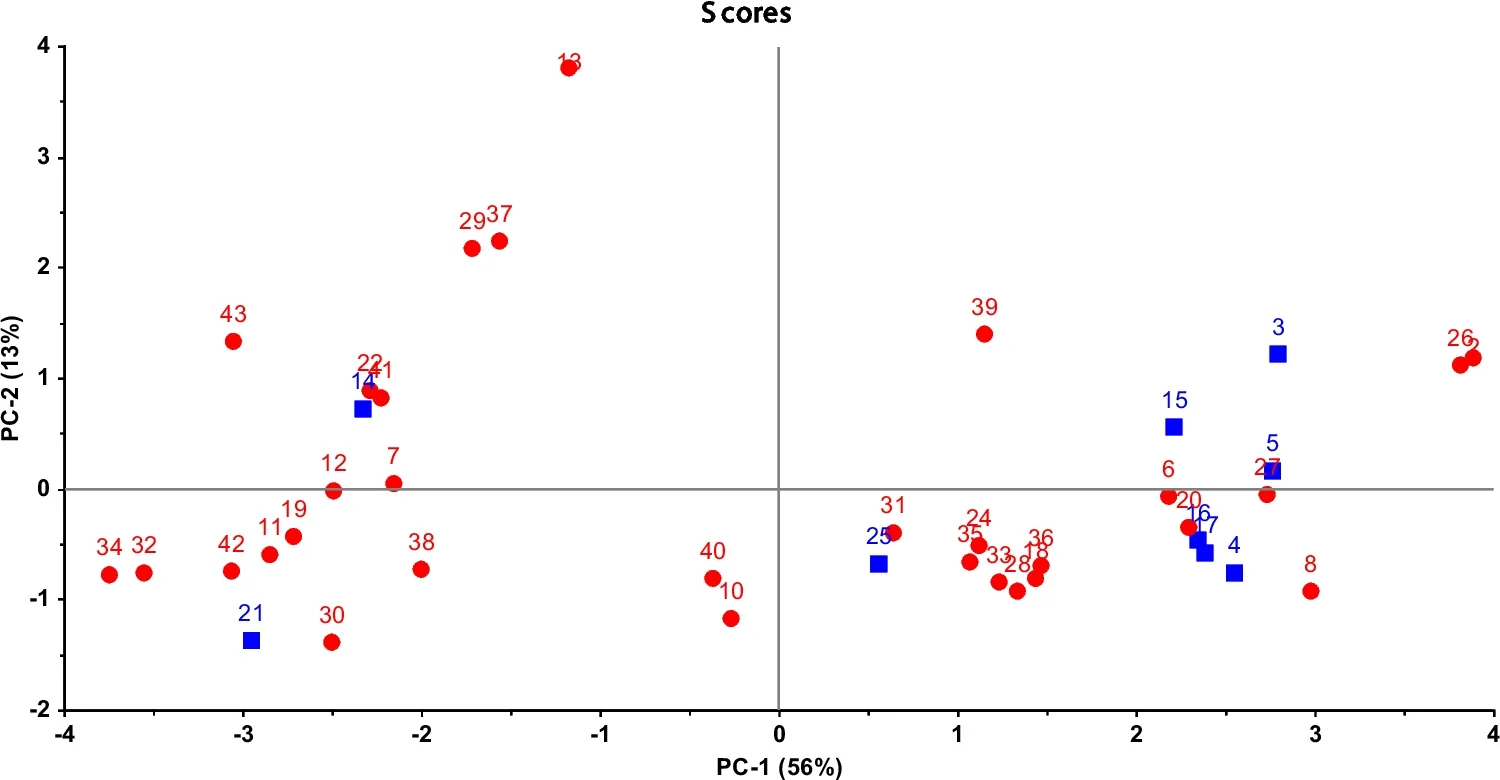
Scores plot of PCA for shell samples specified by fibropapillomatosis: presence (blue boxes) and absence (red dots). PC—principal component. (For interpretation of the references to color in this figure legend, the reader is referred to the web version of this article)

**Fig. 4:**
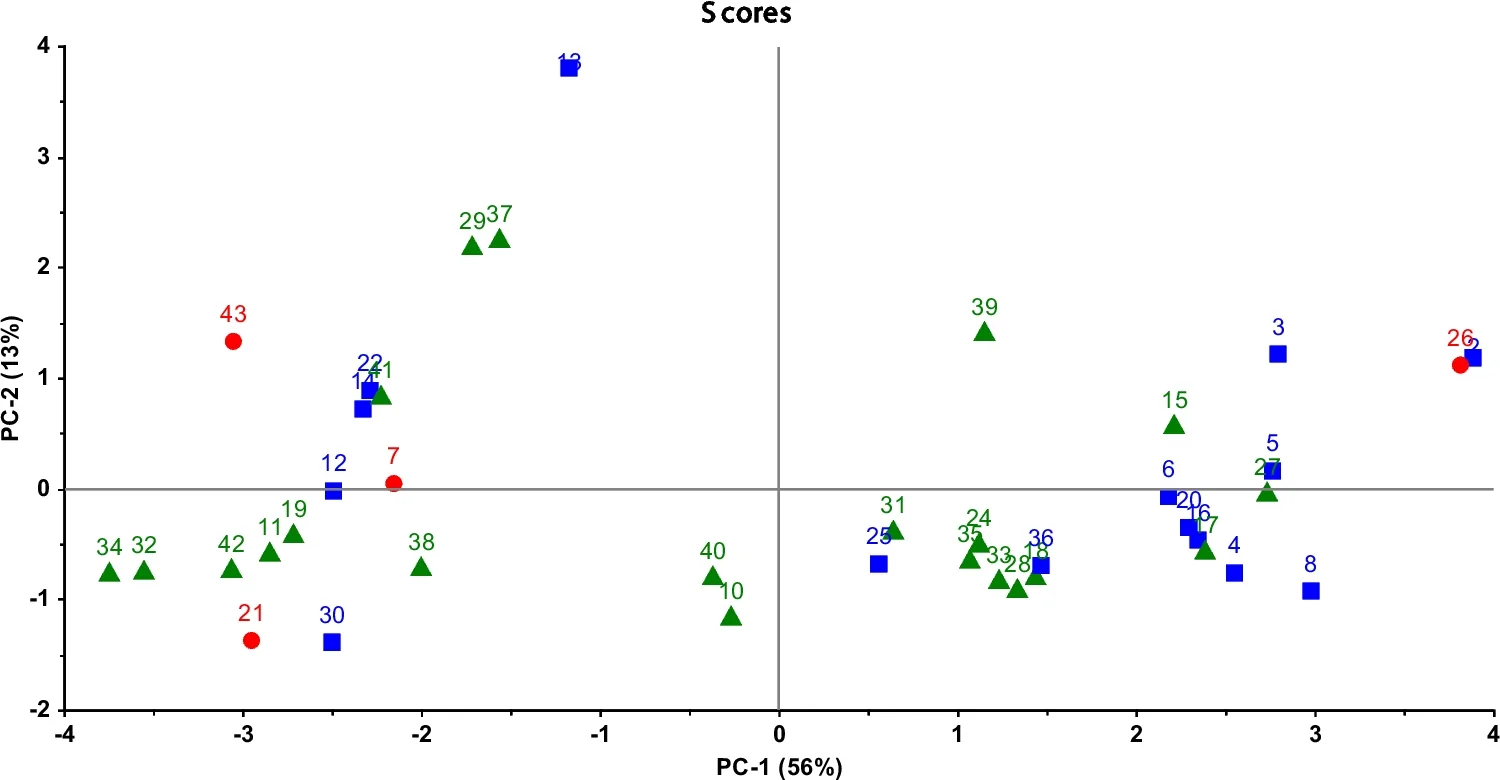
Scores plot of PCA for shell samples specified by location of stranding: area 1 (blue boxes), area 2 (red dots), and area 3 (green triangles). PC—principal component. (For interpretation of the references to color in this figure legend, the reader is referred to the web version of this article)

**Fig. 5:**
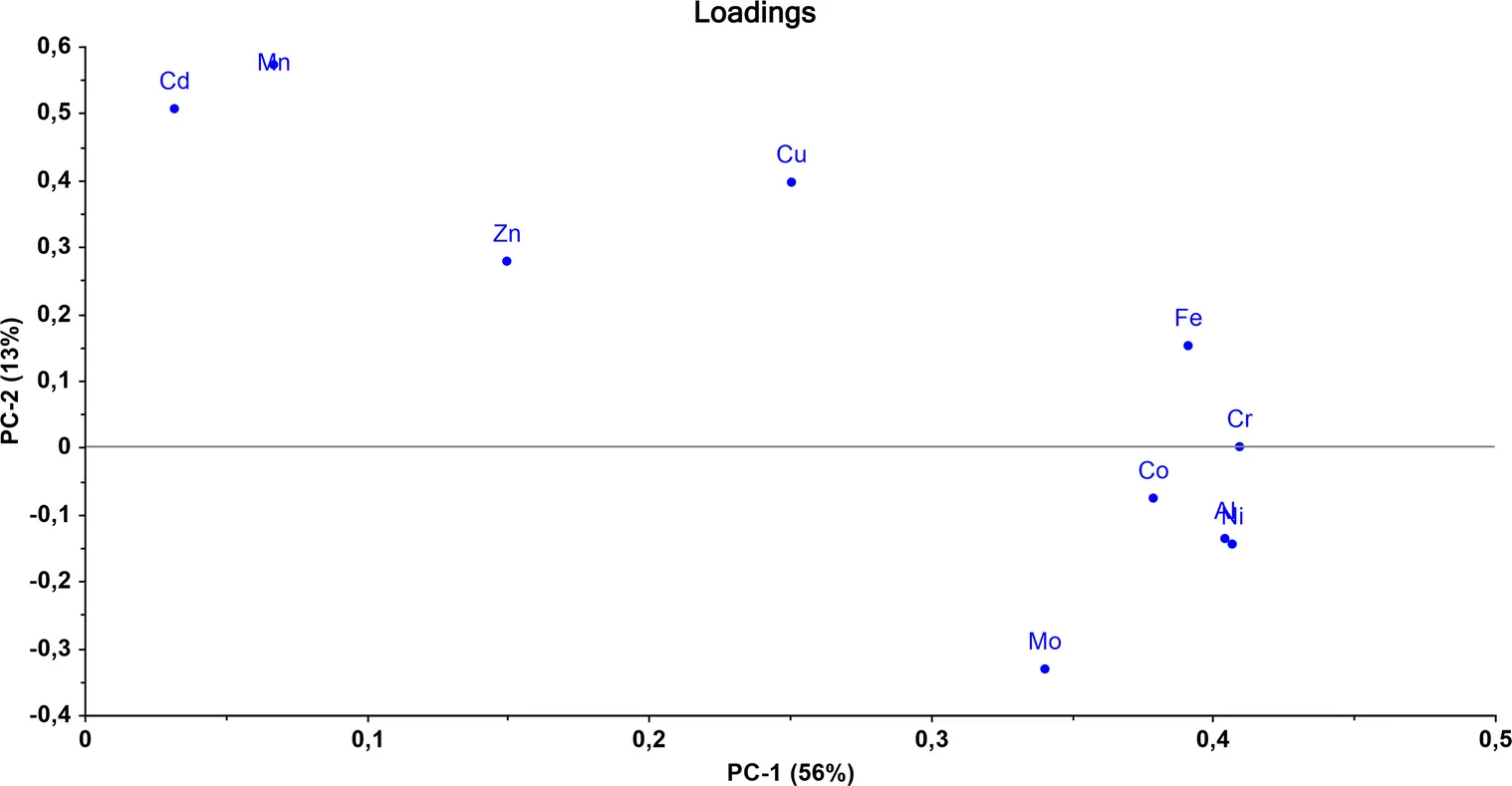
Loadings plot of PCA for shell samples

A PCA was carried out to verify the existence of behavior patterns in the samples, considering the categories evaluated (sex, FP, and stranding area). More relevant results were verified for the shell samples, and the scores plots are shown in Figs. 2, 3, and 4 for the factors sex, FP, and stranding area, respectively. Figure 5 shows the loadings plot for the shell samples.

A preliminary statistical treatment was conducted and outlier samples (codes 1, 9, and 23) were excluded from the PCA. The PCA of the mineral profile of the shell samples provided the explanation of 69% of the variance of the data with the first two PCs, cumulatively.

For the sex category, most samples of male individuals were located to the right along PC1 (Fig. 2). The elements that presented greater loadings in the positive direction of PC1 were Al, Co, Cr, Fe, and Ni (Fig. 5). Consequently, the shells of male individuals tend to have higher mean concentrations for these five metals (Table 3). Furthermore, Fig. 2 shows that the mineral composition of shells of female individuals presented a greater variability than that of males, which can be verified by the wider concentration ranges for most elements (Table 3).

Regarding the FP category, most samples from individuals which presented this disease were located to the right along PC1 (Fig. 3). The elements that presented greater loadings in the positive direction of PC1 were Al, Co, Cr, Fe, and Ni (Fig. 5). Consequently, the shells of individuals affected by FP tend to have higher mean concentrations for these five elements (Table 3). The PCA results complement those obtained via ANOVA. While ANOVA indicated higher Cu concentrations in individuals with FP, PCA proved more sensitive in discriminating between the groups based on Al, Co, Cr, Fe, and Ni concentrations, revealing differences that were not as clearly detected by the univariate analysis.

Finally, for the stranding area category, most samples of individuals from Area 2 (Paiva and Suape beaches) were located to the left along PC1 (Fig. 4). Figure 5 does not show any element with a high loading in that direction of PC1. However, elements Al, Co, Cr, Fe, and Ni have a high loading in the opposite direction. This causes individuals in Area 2 to have the lowest concentrations for these five elements.

A different mineral accumulation trend was verified for the kidney samples. The plots of scores (Figure S1) and loadings (Figure S2) are shown in the Supplementary Material. A preliminary statistical treatment was conducted and outlier samples (codes 5, 9, and 14) were excluded from the PCA. The PCA of the mineral profile of the kidney samples provided the explanation of 57% of the variance of the data with the first two PCs, cumulatively.

For the stranding area category, most samples of individuals from Area 2 were located to the right along PC1 (Figure S1). The elements that presented greater loadings in the positive direction of PC1 were Al, Cr, and Ni (Figure S2). Consequently, the kidneys of individuals from Area 2 tend to have higher mean concentrations for these three elements (Table 6).

The PCA was also performed for the other biological samples studied (liver and muscle), but no relevant behavior pattern was verified in the mineral profile data of these tissues.

## Discussion

### Evaluation of Digestion Efficiency and LOQ

The RCC results were acceptable (up to 35%) when compared to those obtained for other similar materials, such as lean meat (25—30%), bovine liver (24—32%) [62], liver (26.1%), muscle (22.5%), and kidney (35.7%) of a freshwater turtle [63], indicating that the digestion methods employed were efficient. The RCC value for the shell was 34.9%, consistent with keratin-rich biological matrices, such as capybara hair (21.0%) [64].

Obtaining low RCC values is essential for the proper performance of the mineral quantification step, since the introduction of large amounts of non-decomposed organic matter into analytical equipment can cause clogging of the sample introduction system, plasma instability and intensification of background emissions when the ICP-OES technique is used [65].

The achieved OCC values were compared with those available in the literature for different samples, such as capybara hair (44%) [64], bovine liver (51%), lean meat (50%), bovine muscle (41%), pork kidney (49%) [62], liver (66%), muscle (45%), and kidney (47%) of a freshwater turtle [63]. Similar OCC results were verified for identical biological tissues or with equivalent composition even being obtained from animals from completely different taxonomic groups.

### Principal Component Analysis (PCA)

Although the PCA results are informative (Section "Effect of the Sex, Fibropapillomatosis, and Stranding Area on the Mineral Accumulation in Biological Samples"), increasing the sample of (i) male individuals and (ii) specimens from Area 2 (Paiva and Suape) is necessary to strengthen the trends identified in the exploratory analysis. Because sampling relied on carcasses, adequate representation of these two groups was unattainable: only (i) 7 males out of 43 individuals and (ii) 4 from Area 2 were included.

### Mineral Profile of Biological Samples of *C. mydas*

A wide variety of studies have evaluated the concentrations of essential and non-essential elements in *C. mydas* tissues around the world [1, 3, 15, 23, 24, 29, 31–34, 41, 47, 66–86].

Meanwhile, only a few studies have focused on the relationships between adverse biological effects and the concentrations of these elements in turtle tissues. Most studies have investigated the Northeast region [87], but research has also been conducted in other regions of Brazil, including coastal and oceanic waters. Other sites include the Cananéia estuary in the state of São Paulo [88]; the coast of the state of Ceará, Northeast Brazil [68]; Atol das Rocas, Northeast Brazil [89–91]; and the states of Ceará, Maranhão, and Bahia [92]. Further studies have been conducted on the southern coast of the Brazilian Atlantic Ocean [70]; the northern coast of Bahia [74]; Todos os Santos Bay, Bahia [93]; the southern region of the state of Espírito Santo [73]; and in Bahia's coastal area, Brazil's largest loggerhead turtle nesting site [94]. Additional research includes the beaches of Forte, Sauípe, and Camaçari in northern Bahia [95]; Southeast Brazil [96]; Espírito Santo [97]; the Potiguar basin in the state of Rio Grande do Norte [77]; Northeast Brazil [98]; São Paulo [47, 99]; and the states of Maranhão, Ceará, Pernambuco, and Bahia [98].

Like in the present study, other studies have also found no correlation between the concentrations of the elements and the biometric measurements (CCL, CCW, and weight) of the animals. Although size/age can be an important factor in the concentration of elements in tissues [67], other studies have also shown results congruent with ours [19, 20, 47, 68, 75, 78].

Maffucci et al. [20] stated that this lack of correlation is constantly observed when analyzing wild animals, since (1) there is unequal distribution of individuals of the same size; (2) there is an absence of very young or adult specimens, and (3) there is a wide variation in eating habits. Da Silva et al. [47], Bezerra et al. [68], and Jerez et al. [19] stated that in addition to the individuals being at the same level of development, probably the limited number of available samples and the uneven distribution in the body classification are also among the reasons for this lack of correlation.

Although Cu exhibited distinct tissue-specific distributions, the highest concentrations were observed in the liver, followed by the kidneys (Table 7). This pattern has also been reported in several previous studies [2, 27, 41, 67, 70, 72, 78, 88, 100]. Compared with the present study (Table 2), lower hepatic Cu concentrations have been reported in Brazil and other regions [15, 74, 77, 79, 88, 101], whereas higher concentrations were observed in the Northern Hemisphere [15] and other geographically distinct areas [70, 100, 102]. Renal Cu concentrations were comparable to those reported in Japan [102] and along the southern Adriatic coast of Italy [101], but exceeded those recorded in the Potiguar Basin, northeastern Brazil [77]. In contrast, higher renal concentrations have been described in China [100], on the northeastern coast of Bahia [74], in the Cananéia Estuary [88], and on the South Atlantic coast of Brazil [70]. For muscle tissue, only lower Cu concentrations have been reported, particularly in turtles from the Potiguar Basin, northeastern Brazil [77] (Table 7).

**Table 7:** Cu concentrations (mg kg⁻^1^) in liver, muscle, and kidneys of green turtles (*Chelonia mydas*) compared with studies conducted in different locations

Tissue	Mean and/or concentration range (mg kg⁻^1^)	Study site	Reference
Liver	67.45	Northern Hemisphere	Fraga et al. [15]
Liver	16.79	Southern Hemisphere	
Liver	100.9 ± 15.9	Southern Atlantic, coast of Brazil	Silva et al. [70]
Kidney	12.2 ± 1.1		
Liver	36.70	Northern coast of Bahia, Brazil	Macêdo et al. [74]
Kidney	13.6 ± 6.5		
Liver	25.11	Potiguar Basin, Northeastern Brazil	Farias et al. [77]
Kidney	1.75		
Muscle	0.24		
Liver	31.52	State of Espírito Santo, Brazil	Tostes et al. [79]
Liver	39.90	Cananéia estuary, Brazil	Barbieri [88]
Liver	20.70		
Kidney	12.55 ± 1.04		
Liver	133 ± 149	South Waters, China	Lam et al. [100]
Kidney	15.0 ± 7.2		
Liver	32.75	Italian coast of the South Adriatic Sea	Storelli et al. [101]
Kidney	8.20 ± 4.20		
Liver	139 ± 86	Yaeyama Islands, Japan	Anan et al. [102]
Kidney	8.27 ± 4.06		
Liver	65 (6.4–212)	South Atlantic, Northeast Brazil	This study
Kidney	8.4 (3.2–38)		
Muscle	1.5 (< 0.21–35)		
Shell	2.7 (0.59–7.8)		

Yipel et al. [72] and Andreani et al. [67], when analyzing metals in *C. mydas* and *Caretta caretta* (loggerhead turtle), found surprisingly that Cu was one of the few metals with higher concentrations in green turtles than in loggerhead turtles*.* The authors argued that the reason was the herbivorous feeding habit of the species, since the tissues with highest Cu (blood and liver) are tissues that reflect recent exposure to the elements [8, 31, 41]. Thus, macroalgae and seagrass bioaccumulate this and other toxic elements [103, 104], so eating habit is the main hypothesis for the accumulation of Cu in the tissues of *C. mydas* [25, 67, 78, 105, 106].

As for Cd, the tissues with the highest concentrations were the kidneys and liver, whereas markedly lower concentrations were observed in muscle and carapace (Table 2). This distribution reflects the organotropism commonly observed for Cd in vertebrates, in which specific organs preferentially accumulate certain elements [107]. In the present study, the kidneys exhibited the highest Cd concentration (49 mg kg⁻^1^), followed by the liver (12 mg kg⁻^1^) and muscle (0.29 mg kg⁻^1^), indicating that Cd accumulation was concentrated primarily in the renal and hepatic tissues. This tissue distribution suggests prolonged Cd exposure in the studied population of *C. mydas* from the southern coast of Pernambuco. The predominance of Cd in the kidneys over the liver is consistent with the progressive redistribution of this element within the organism during chronic exposure, resulting in preferential accumulation in organs involved in metal storage and excretion.

The tissue distribution observed in the present study is consistent with previous reports showing that Cd preferentially accumulates in the kidneys and liver of sea turtles (Table 8). Guirlet and Das [108] and Sinaei et al. [76] reported that turtles exposed to Cd through food accumulate this element differentially among tissues, with the highest concentrations occurring in the kidneys, followed by the liver, pancreas, and muscle. Likewise, the liver and kidneys have consistently been identified as the principal organs of trace metal accumulation in sea turtles [109]. The distribution pattern observed in the present study, with the kidneys presenting the highest Cd concentration, followed by the liver and muscle, agrees with previous findings reported by Guirlet and Das [108], Nicolau et al. [110], and Abdallah [111]. Furthermore, Nordberg et al. [112] demonstrated that the liver and kidneys together retain approximately half of the total body Cd burden, while Guirlet and Das [108] observed that Cd accumulation in turtle tissues occurs in a dose-dependent manner.

**Table 8:** Cd concentrations (mg kg⁻^1^) in liver, muscle, kidneys, and shell of green turtles (*Chelonia mydas*) compared with studies conducted in different locations

Tissue	Mean and/or concentration range (mg kg⁻^1^)	Study site	Reference
Liver	0.01 ± 0.02	Saudi Arabia’s largest rookery	Tanabe et al. [12]
Kidney	0.01 ± 0.02		
Muscle	0.11 ± 0.24		
Liver	0.05–1.88	Mediterranean coastline of Murcia, Spain	Jerez et al. [19]
Kidney	0.02–16.16		
Muscle	0.08 ± 0.06 (0.02–0.24)		
Liver	8.96 (3.21–21.6)	Japão	Anan et al. [24]
Liver	1.20 (0.12–5.18)	Baja California peninsula, Mexico	Gardner et al. [27]
Kidney	121 (6.1–653)		
Muscle	0.01 (ND–39.2)		
Liver	16.92 (ND–72.6)	Baja California peninsula, Mexico	Talavera-Saenz et al. [41]
Kidney	110 (65.1–653)		
Liver	0.13 (0.05–0.46)	Brazilian river (Females)	da Fonseca et al. [63]
Liver	0.15 (0.05–0.7)	Brazilian river (Males)	
Kidney	0.26 (< 0.084–0.7)	Brazilian river (Females)	
Kidney	0.28 (< 0.084–0.9)	Brazilian river (Males)	
Muscle	< 0.053	Brazilian river (Females)	
Muscle	0.11 (< 0.053–1.9)	Brazilian river (Males)	
Liver	10.6 ± 1.1	North Caribbean coast, Costa Rica	Andreani et al. [67]
Kidney	39.2 ± 3.1		
Liver	5.9 ± 0.9 (0.6–25.4)	Southern Atlantic coast of Brazil	Silva et al. [70]
Kidney	28.3 ± 2.3 (2.1–51.9)		
Muscle	0.4 ± 0.1 (0.02–1.7)		
Liver	0.5 ± 0.31	Northeast Mediterranean Sea	Yipel et al. [72]
Kidney	4.24 ± 1.01		
Liver	66.0 ± 0.05	Northern coast of the Sea of Oman	Sinaei et al. [76]
Kidney	36.00 ± 0.02		
Muscle	33 ± 0.02		
Liver	5.71 ± 4.35 (1.10–20.82)	Potiguar Basin, northeastern Brazil	Farias et al. [77]
Kidney	16.06 ± 0.13		
Muscle	0.072 ± 0.129		
Liver	3.3 (ND–102)	Campos and Espírito Santo basins, Brazil	Tostes et al. [79]
Liver	0.3 ± 0.1	Cananéia estuary, Brazil (Juveniles)	Barbieri [88]
Liver	1.0 ± 0.3	Cananéia estuary, Brazil (Adults)	
Kidney	1.0 ± 0.3	Cananéia estuary, Brazil (Juveniles)	
Kidney	2.2 ± 0.3	Cananéia estuary, Brazil (Adults)	
Liver	1.0 ± 0.3	Southeastern Brazil	Bruno et al. [96]
Kidney	24.4 ± 10.6		
Liver	1.5 ± 0.6	South Waters, China	Lam et al. [100]
Kidney	2.5 ± 1.8	South Waters, China	
Muscle	ND–0.096	South Waters, China (Juveniles)	
Muscle	0.17 ± 0.06	South Waters, China (Adults)	
Liver	4.66 ± 2.33 (0.32–10.56)	Italian coast of the South Adriatic Sea	Storelli et al. [101]
Kidney	5.1 (2.2–7.5)		
Liver	18.2 ± 9.7	Yaeyama Islands, Japan	Anan et al. [102]
Kidney	142 ± 64		
Muscle	0.24 ± 0.17		
Liver	5.58 ± 4.05 (0.30–18.6)	Yaeyama Islands, Okinawa, Japan	Sakai et al. [106]
Kidney	38.5 ± 21.3 (7.3–80.7)		
Muscle	0.05 ± 0.08 (0.01–0.54)		
Liver	5.03 ± 0.54	Coast of mainland, Portugal	Nicolau et al. [110]
Kidney	34.67 ± 3.21		
Muscle	0.16 ± 0.01		
Liver	23.82 (1.77–118.18)	Hawaiian Islands, USA	Aguirre et al. [113]
Kidney	56.79 (16.86–250.71)		
Liver	12.5 (2.5–56.9)	Australia	Gordon and Pople [114]
Liver	5.89 (2.53–10.73)	Eastern Mediterranean Sea	Godley et al. [115]
Kidney	30.5 (18.8–42.2)		
Kidney	27.33 ± 25.44	Mediterranean coast of Turkey (Female)	Simsek et al. [116]
Kidney	25.28 ± 23.81	Mediterranean coast of Turkey (Male)	
Muscle	0.18 ± 0.07	Mediterranean coast of Turkey (Female)	
Muscle	4.77 ± 10.16	Mediterranean coast of Turkey (Male)	
Liver	0.90 ± 0.13	Padre Island National Seashore, USA	Faust et al. [117]
Kidney	3.07 ± 0.61		
Muscle	0.043		
Shell	0.016	Hawaii, USA (Captivity)	Shaw et al. [4]
Shell	0.014	Hawaii, USA (Wild)	
Shell	0.1 ± 0.03	Toolakea Beach, Australia	Wilkinson et al. [14]
Shell	0.05 ± 0.04	Upstart Bay, Australia	
Shell	0.07 ± 0.03	Cockle Bay, Australia	
Shell	0.07 ± 0.03	Edgecumbe Bay, Australia	
Shell	0.30 ± 0.06	San Diego, USA	Barraza et al. [75]
Shell	0.09 ± 0.08	Cape Cod Bay, Massachusetts, USA	Chan et al. [82]
Shell	0.008 (0.0002–0.05)	Southeast Brazil	Miguel et al. [97]
Liver	12 (3.5–44)	South Atlantic, Northeast Brazil	This study
Kidney	49 (8.7–177)		
Muscle	0.29 (< 0.053–4.6)		
Shell	0.15 (< 0.14–0.19)		

*ND* Not detected

The Cd concentrations measured in this study are generally consistent with those reported for *C. mydas* worldwide (Table 8). However, Brazilian specimens exhibited comparatively higher concentrations in the liver, kidneys, and muscle than those reported in most previous national studies.

In liver tissue, the Cd concentrations observed in the present study (12 [3.5–44] mg kg⁻^1^) fall within the range reported for the species in several geographic regions, including the Mediterranean Sea, Japan, China, Mexico, Costa Rica, Brazil, Portugal, and Iran [19, 24, 27, 67, 70, 72, 77, 79, 88, 96, 100, 101, 106, 110, 115, 117] (Table 8). Higher mean hepatic concentrations have been reported in the USA [113], Japan [102], Mexico [41], and Iran [76], whereas Gordon et al. [114] reported values similar to those found in the present study.

Renal Cd concentrations in the present study (49 [8.7–177] mg kg⁻^1^) exceeded those reported in many previous investigations, although higher values have been described in populations from the USA [113], Japan [102], Mexico [27, 41], and other locations [67, 70, 72, 76, 96, 100, 101, 106, 110, 114–117] (Table 8). Similarly, muscle Cd concentrations were generally higher than those reported in the literature, except for the markedly elevated values described by Sinaei et al. [76]. For the carapace, Cd concentrations exceeded those reported in most previous studies [4, 14, 75, 82, 97], although Barraza et al. [75] reported significantly higher concentrations in turtles from San Diego (USA).

Talavera-Saenz et al. [41] likewise reported unusually high hepatic and renal Cd concentrations in *C. mydas* compared with other populations worldwide (Table 8). According to these authors, tissue Cd concentrations primarily reflect environmental exposure because Cd uptake is not actively regulated, and its distribution among organs depends largely on both the duration and intensity of exposure. Therefore, the elevated tissue concentrations observed in the present study are consistent with previous reports [20, 27, 31, 41, 76, 102, 105, 113, 118] and most likely reflect the progressive accumulation of Cd throughout the lifespan of this long-lived species. Moreover, Talavera-Saenz et al. [41] and Anan et al. [102] proposed that renal Cd concentrations provide a reliable indicator of long-term exposure, whereas hepatic concentrations more accurately reflect recent dietary exposure.

The preferential accumulation of Cd in the kidneys and liver can be explained by the physiological mechanisms governing Cd transport, storage, and detoxification. During prolonged exposure, Cd initially accumulates in the liver, where it binds to metallothionein (MT), a low-molecular-weight metal-binding protein involved in the detoxification of toxic metals and the homeostasis of essential elements [67]. The MT synthesis is particularly induced in organs associated with metal uptake, storage, and excretion, such as the intestine, liver, and kidneys [31]. Subsequently, the Cd–MT complex is released into the bloodstream and transported to the kidneys, where Cd progressively bioaccumulates to concentrations exceeding those in the liver [112, 119]. This transport pathway explains the predominance of Cd in renal tissue observed in the present study.

The progressive redistribution of Cd from the liver to the kidneys is characteristic of chronic exposure because Cd elimination is extremely limited, resulting in prolonged retention, particularly in renal tissue [111]. Consequently, renal Cd concentrations are considered reliable indicators of long-term exposure, whereas hepatic concentrations more closely reflect recent dietary intake [41, 102]. The elevated Cd concentrations observed in the present study are therefore compatible with chronic environmental exposure and the long lifespan of *C. mydas*, which favors gradual accumulation over time.

Several factors may contribute to the variability of Cd concentrations among individuals and populations. Environmental contamination, differences in trophic exposure, age, and feeding ecology have all been proposed as important determinants of tissue Cd concentrations. Because Cd accumulates throughout life, age-related dietary shifts may influence exposure patterns. Juvenile green turtles inhabiting coastal environments may accumulate higher Cd concentrations through the ingestion of zooplankton and other low-trophic-level prey [111, 119]. In addition, Fraga et al. [119] suggested that large-scale natural and anthropogenic Cd sources may exert a stronger influence on Cd concentrations in marine organisms than local contamination sources. Consistent with this hypothesis, higher dissolved Cd concentrations have been reported in the Pacific Ocean than in the Atlantic Ocean, indicating that global-scale processes may substantially influence Cd availability in marine ecosystems.

Although MT induction is generally considered a protective mechanism against Cd toxicity, the Cd–MT complex is itself nephrotoxic, providing a mechanistic explanation for the preferential renal accumulation of this element following chronic exposure [70, 120, 121].

The elevated Cd concentrations observed, particularly in the kidneys of *C. mydas*, represent one of the most important toxicological findings of the present study. Sea turtles are known to accumulate unusually high Cd concentrations in several regions worldwide [41], and the concentrations measured in the present study are consistent with this global pattern, although they are comparatively higher than those reported in most previous Brazilian studies.

Despite the high Cd concentrations found in the liver and kidneys, information regarding the toxic effects of Cd in *C. mydas* remains limited, making it difficult to establish biologically meaningful threshold values for this species. No studies have yet demonstrated a direct relationship between renal Cd accumulation and kidney dysfunction in sea turtles, nor have toxic limits been established specifically for turtles. Nevertheless, studies in other vertebrates indicate that Cd is highly toxic, with toxic hepatic and renal concentrations reported for ruminants (50–160 mg kg^−1^ and 100–200 mg kg^−1^, respectively) [120] and maximum permissible concentrations established for terrestrial mammals by Brazilian legislation. The liver and kidney concentrations measured in the present study exceeded these regulatory limits, emphasizing the remarkable Cd burden accumulated by the studied turtles.

Experimental evidence also suggests that juvenile green turtle liver cells are comparatively more resistant to Cd toxicity than mammalian and fish cells, indicating that these cells may serve as useful biological models for evaluating mixed-metal contamination and potential risks to wildlife and human health. Furthermore, because the liver plays a central role in metal homeostasis, identifying tissues that can be sampled non-destructively while reliably reflecting trophic exposure remains an important challenge for sea turtle biomonitoring.

The elevated Cd concentrations observed in the present study are likely associated with chronic environmental exposure, contamination of coastal ecosystems, ingestion of contaminated food, and the long-term bioaccumulation characteristic of this species. Consequently, further studies addressing contaminant transfer through food webs, ontogenetic dietary shifts, and exposure dynamics across different life stages are needed to better understand the factors controlling Cd accumulation and its consequences for sea turtle health.

Overall, the Cd concentrations detected in the liver, kidneys, and muscle highlight the need for continued monitoring of this element in *C. mydas*. Given that Cd is recognized among the most toxic environmental trace elements, these findings reinforce the importance of long-term ecotoxicological monitoring programs to improve risk assessment and support conservation strategies for sea turtle populations inhabiting the southern coast of Pernambuco.

The results of the present study indicate that Al concentrations in turtles stranded along the southern coast of Pernambuco are higher than those reported for *C. mydas* from other regions [74, 75, 79, 96, 121, 122]. Previous studies in California (USA) showed that variations in Al concentrations reflected local sediment characteristics, suggesting that this element may bioaccumulate through the turtles' diet [75] (Table 9).

**Table 9:** Al concentrations (mg kg⁻^1^) in liver, muscle, kidneys, and shell of green turtles (*Chelonia mydas*) compared with studies conducted in different locations

Tissue	Mean and/or concentration range (mg kg⁻^1^)	Study site	Reference
Liver	20.2 ± 21.7 (6.9–58.4)	Northern coast of Bahia, Brazil	Macêdo et al. [74]
Kidney	69.6 ± 57.7 (13.8–179)		
Shell	318.62 ± 63.71 (14.62–829.64)	Seal Beach National Wildlife Refuge, California, USA	Barraza et al. [75]
Shell	209.99 ± 40.18 (12.44–627.79)	San Diego Bay, California, USA	
Liver	4.47 ± 2.37	State of Espírito Santo, Brazil	Tostes et al. [79]
Kidney	5.321 ± 5.39	Rio de Janeiro, Brazil (Infants)	Bruno et al. [96]
Kidney	2.893 ± 0.85	Rio de Janeiro, Brazil (Juveniles)	
Muscle	2.217 ± 0.49	Rio de Janeiro, Brazil (Infants)	
Muscle	7.745 ± 13.32	Rio de Janeiro, Brazil (Juveniles)	
Liver	2.21 (0.12–31.08)	Canary Islands, Spain	Torrent et al. [121]
Kidney	13.80 (1.16–122.12)		
Muscle	7.35 (1.55–67.22)		
Kidney	10–51	Florida, USA	Homer et al. [122]
Liver	441 (< 4.0–928)	South Atlantic, Northeast Brazil	This study
Kidney	644 (248–808)		
Muscle	< LOQ		
Shell	1064 (148–1865)		

*LOQ* Limit of quantification

A few studies have investigated the nature and composition of essential and non-essential elements in food items for green turtles obtained from the collection area or nearby regions. Rodrigues et al. [123] recorded that the diet of *C. mydas* consisted mainly of red algae, especially the species *Pterocladiella beachiae* (65.90% of occurrence), followed by rhodophytes as well as Gelidiales, *Gelidium lineare* (52.27%), and *Gelidiella acerosa* (47.72%). In addition, some items of animal origin were also found, such as the soft coral *Palythoa caribaeroum* (first record for this region), the planktonic mollusks *Jhantina jhantina* and *Spirula* sp., as well as the sponges *Geodia gibberosa* and *Steletta* sp. The contents of the digestive tract showed a strong herbivorous pattern, with evidence suggesting that green turtles feed mainly on marine macroalgae. It is important to consider that the feeding habits of *C. mydas* during the pelagic phase are almost exclusively carnivorous, whereas in the coastal phase they feed more on marine algae and grasses, which may explain why strandings are more frequent in juvenile *C. mydas* than in adults.

Brito et al. [124] determined the concentrations of some minerals in macroalgae species in Bahia and reported high levels of As, Cd, Co, Mn, Ni, Pb, and Zn. Therefore, the high concentrations of Al found in our studied region might be associated with disturbance of sediments contaminated by this element, which can remobilize contaminants linked to sediments in the water column, where they potentially impact a wide range of species and are incorporated into trophic networks [125]. Komoroske et al. [66] showed that different types of algae present significant differences in the patterns of absorption and bioaccumulation of elements, and suggested that for the analyzed region, macroalgae, the main components of the *C. mydas* diet [123], must be accumulating high levels of Al.

Some elements, such as Co, Mo, Mn, and Ni, despite posing numerous risks to organisms [33], are rarely used in biomonitoring studies. Ley-Quiñónez et al. [29] emphasized that Ni is little studied in investigations of sea turtles, and the general levels reported are below the detection limits [27, 31, 106]. In our study, Ni concentrations were higher than those reported in previous studies [27, 31, 74, 106, 126] (Table 10). For specimens both with and without FP, da Silva et al. [47], studying the population of Ubatuba (São Paulo), reported higher concentrations of Ni than those found in this study.

**Table 10:** Ni concentrations (mg kg⁻^1^) in liver, muscle, kidneys, and shell of green turtles (*Chelonia mydas*) compared with studies conducted in different locations

Tissue	Mean and/or concentration range (mg kg⁻^1^)	Study site	Reference
Liver	0.01 (ND-7.40)	Baja California peninsula, Mexico	Gardner et al. [27]
Kidney	1.15 (ND-26.43)		
Muscle	0.03 (ND-4.0)		
Liver	< 0.03	Coastal waters, Japan	Sakai et al. [31]
Kidney	0.217 ± 0.093		
Muscle	0.083 ± 0.026		
Shell	0.094 ± 0.022		
Blood	44.07 ± 5.83 (FP tumor score 0)	Ubatuba coast, São Paulo, Brazil	Da Silva et al. [47]
	46.74 ± 4.60 (FP tumor score 1)		
	57.85 ± 13.79 (FP tumor score 2)		
	50.01 ± 8.84 (FP tumor score 3)		
Liver	0.79 ± 0.34	Northern coast of Bahia, Brazil	Macêdo et al. [74]
Kidney	1.92 ± 1.41		
Liver	0.06–0.31	Yaeyama Islands, Okinawa, Japan	Sakai et al. [106]
Kidney	0.615 ± 0.36 (0.124–1.33)		
Liver	0.28 ± 0.08	Rio de Janeiro, Brazil	Barbieri [126]
Kidney	0.19 ± 0.02		
Liver	4.4 (< 2.2–11)	South Atlantic, Northeast Brazil	This study
Kidney	5.9 (< 2.2–8.1)		
Muscle	16 (< 0.46–31)		
Shell	13 (< 2.2–27)		

*FP* Fibropapillomatosis; *ND* Not detected

Labrada-Martagón et al. [127], in Punta Abreojos (Mexico), found high concentrations of Ni in the blood of green turtles, reflecting recent environmental exposure to this essential element, and consequently, oxidative stress, also recent. The data reported in our study show that in all tissues analyzed, Ni maintained an average above 4.4 mg kg^−1^ (dry mass), showing highest values in muscle (mean of 16 mg kg^−1^) and shell (mean of 13 mg kg^−1^), demonstrating recent exposure to this element [10]. The toxicity of Ni in organisms depends on its concentration in the water and the organism's physiology [128]. Thus, despite the low affinity and low concentrations in marine vertebrates [29, 129, 130], further research is needed to investigate the role of Ni in these organisms, given its immunosuppressive toxicity in animals [131] and the precariousness of studies involving this element and tropical marine species [132].

The carapace exhibited the highest Mn concentrations, followed by the liver and kidneys (Table 2). The concentrations observed in the present study were substantially higher than those reported by Sakai et al. [106] and Talavera-Saenz et al. [41], but were comparable to those found in *C. mydas* by Andreani et al. [67] and in *C. caretta* by Franzellitti et al. [25]. Although Mn concentrations have also been investigated in *C. mydas* from other regions [74, 79, 96, 126], available data remain limited. According to Ley-Quiñónez et al. [29], this may reflect either low environmental exposure or the relatively rapid elimination of Mn through the respiratory and digestive tracts (Table 11).

**Table 11:** Mn concentrations (mg kg⁻^1^) in liver, muscle, and kidneys of green turtles (*Chelonia mydas*) compared with studies conducted in different locations

Tissue	Mean and/or concentration range (mg kg⁻^1^)	Study site	Reference
Liver	6.23 ± 2.80	Northwestern Adriatic Sea, Italy	Franzellitti et al. [25]
Muscle	2.7 ± 1.2		
Liver	0.24 (ND-5.31)	Baja California península, Mexico	Talavera-Saenz et al. [41]
Kidney	1.51 (ND-7.73)		
Liver	8.92 ± 0.93	North Caribbean coast, Costa Rica	Andreani et al. [67]
Kidney	5.75 ± 0.28		
Liver	8.73 ± 2.45	Northern coast of Bahia, Brazil	Macêdo et al. [74]
Kidney	6.05 ± 2.81		
Liver	1.9 ± 0.58	State of Espírito Santo, Brazil	Tostes et al. [79]
Kidney	1.345 ± 0.763	Southeastern Brazil	Bruno et al. [96]
Muscle	0.148 ± 0.503		
Liver	2.18 ± 0.40	Yaeyama Islands, Okinawa, Japan	Sakai et al. [106]
Kidney	1.50 ± 0.51		
Muscle	0.28 ± 0.11		
Liver	4.32 ± 0.71	Rio de Janeiro, Brazil	Barbieri [126]
Kidney	4.17 ± 0.86		
Liver	7.0 (0.42–17)	South Atlantic, Northeast Brazil	This study
Kidney	5.6 (2.9–12)		
Muscle	0.36 (< 0.099–5.6)		
Shell	17 (1.5–172)		

*ND* Not detected

Studies of toxicity parameters, such as effective and lethal concentration, as well as chronic values of Co in marine organisms are scarce [133]. In our study, the concentrations of Co were low for all tissues, with a slightly higher concentration in kidney tissue. Its toxicity in vertebrates is not well understood, but high exposure to this element is associated with oxidative stress, clinical and hematological markers of stress (renal dysfunction), and inflammatory responses in *C. mydas* [32, 134].

Low affinity of *C. mydas* for Mo was observed, which indicates that this metal apparently has no impact on the health of the population studied. Villa et al. [33] reported absence of toxicokinetic information about Mo in green turtles. This metal helps in the regulation of other metals, such as Cu and Hg, and is part of the composition of several enzymes necessary for diverse stages of metabolism [135]. However, in high concentrations it can be toxic or result in kidney problems [135, 136].

As for Pb, the levels were below the limit of quantification, or had insignificant concentrations, in all tissues analyzed, corroborating other studies [27, 41, 67, 70, 74, 100, 115] (Table 12). Our results differ from those of Sakai et al. [31], because contrary to what they reported, the bone tissue of the turtles analyzed in the south of Pernambuco was not important in the accumulation of Pb. The only tissue that contained a concentration above the limit of quantification for this element was muscle. The Pb concentrations in muscle tissue can be particularly high after prolonged exposure at a high level [31]. In our study, muscle concentrations were not high, suggesting the absence of pronounced exposure to this metal. The individuals found in stranding area 2 exhibited slightly lower Pb concentrations (2.7 mg kg^−1^, Table 5), but this pattern is possibly linked to the lower sample representativeness in that area (*N* = 4).

**Table 12:** Pb concentrations (mg kg⁻^1^) in liver, muscle, kidneys, and shell of green turtles (*Chelonia mydas*) compared with studies conducted in different locations

Tissue	Mean and/or concentration range (mg kg⁻^1^)	Study site	Reference
Liver	ND	Baja California peninsula, Mexico	Gardner et al. [27]
Kidney	0.01 (ND-0.36)		
Muscle	0.01 (ND-1.23)		
Liver	0.08 ± 0.03	Coastal waters, Japan	Sakai et al. [31]
Kidney	0.16 ± 0.05		
Muscle	0.02 ± 0.03		
Shell	2.42 ± 0.52		
Liver	0.05 (ND-1.74)	Baja California península, Mexico	Talavera-Saenz et al. [41]
Kidney	0.00 (ND-0.07)		
Liver	0.152 ± 0.043	South Waters, China	Lam et al. [100]
Kidney	0.311 ± 0.189		
Muscle	0.082 ± 0.103		
Liver	ND-1.84	Eastern Mediterranean Sea	Godley et al. [115]
Kidney	1.81		
Muscle	ND-2.45		
Liver	0.07 ± 0.014	North Caribbean coast, Costa Rica	Andreani et al. [67]
Kidney	0.044 ± 0.002		
Liver	4.5 ± 0.5 (0.9–14.5)	Southern Atlantic coast, Brazil	Silva et al. [70]
Kidney	5.4 ± 0.4 (1.3–10.3)		
Muscle	4.2 ± 0.3 (2.7–8.7)		
Liver	0.53 ± 0.45 (0.18–1.58)	Northern coast of Bahia, Brazil	Macêdo et al. [74]
Kidney	0.15 ± 0.14 (0.03–0.48)		
Liver	< LOQ	South Atlantic, Northeast Brazil	This study
Kidney	< LOQ		
Muscle	4.1 (< 1.4–7.4)		
Shell	< LOQ		

*LOQ* Limit of quantification; *ND* Not detected

Since there is no maximum reference limit in Brazilian legislation to determine the toxic concentrations of elements in sea turtles, food standards can be used for comparison. These are set by Resolution 722/2022 and Normative Instruction 160/2022 from the Brazilian Sanitary Surveillance Agency (ANVISA) [137, 138], which specifies maximum limits of inorganic contaminants in human food, based on fish muscle (Cd 0.05 mg kg^−1^, Pb 0.30 mg kg^−1^). Thus, for Cd, all values found in this study markedly exceeded the permitted limits, and for Pb, the muscle concentration also exceeded the maximum permitted limit.

Silva et al. [70] and da Silva et al. [47] studied metal contamination as a possible etiology of FP in *C. mydas* in Brazil. They found that metal contamination, especially by Cu, Fe, and Pb, may be linked to the disease, as turtles severely affected by FP had significantly higher levels of these metals than those without FP. The authors argued that contamination by these elements could implicate the etiology of FP by generating oxidative stress, although knowledge of oxidative stress in sea turtles remains incipient.

With the exception of Pb, high concentrations of Cu in the liver and Fe in the shell, liver, and kidneys were noteworthy (Table 2). These results corroborate that individual and environmental factors play a significant role in the epidemiology and severity of FP in *C. mydas* [53]. It is worth noting that Fe and Cu can undergo redox cycling and generate reactive oxygen species, which can cause health problems, including tumor formation in various tissues of the animal organism.

Environmental factors can play an important role in the development of FP. These include (i) chemical contaminants from domestic sewage, which pollute coastal waters and sediments with metals; (ii) low immunocompetence; (iii) regions affected by agriculture, industry, and urban development; and (iv) consumption of macroalgae, trace metals, organic pollutants, and natural biotoxins, among others. The influence of marine pollution on the survival and health of sea turtles is not yet well established [46]. Foley et al. [44] found the highest prevalence of FP in *C. mydas* in coastal areas with poor ecological conditions and in expansive, shallow waters.

More recently, Manes et al. [49] reported that seawater temperature, salinity, human population density, and coastal river flow may be important factors in the prevalence of FP. This finding highlights the multifactorial nature of this disease and, despite consensus and assumptions in the literature, little is still known about the specific roles of certain environmental factors in FP [46, 49, 50].

The present study found a significant association between Cu in the shell and the presence of FP. Although Cu and Fe are essential elements for green turtles, high amounts can lead to adverse biological effects [47]. The high concentrations of Cu and Fe found in this study, along with evidence linking these metals to the etiology and development of FP [47], suggest these metals are affecting the health of the *C. mydas* population along the coast of Northeast Brazil.

Zinc plays an important role in the regulation of homeostatic processes, and is considered essential in small concentrations [20, 139]. Macêdo et al. [74], when studying the population of *C. mydas* in northern Bahia, reported concentrations higher than that of this study (132 mg kg^−1^ in liver and 151 mg kg^−1^ in kidney) and suggested that these were not sufficient to cause a toxic effect in the analyzed sea turtles. The high concentrations of Zn in these studies may be the result of the foraging areas of *C. mydas*, since Zn reportedly can be acquired through the food chain [29, 41, 105, 118]. Jakimska et al. [139] suggested that this metal is associated with the synthesis of metalloproteins, acting to protect against the toxic effects of non-essential elements.

Potential differences in foraging behavior, as well as variable signs of exposure during the pelagic phase, may explain the often-greater variability of toxic elements found in juvenile green turtles [32].

Size and age are significant sources of variation in tissue concentrations of toxic elements. Age should have a particularly strong influence for accumulation of metals with a high biological half-life, especially in long-lived species, as long as individuals can survive over the typical lifetime with low exposure levels. Therefore, it is possible to predict higher levels of Cd, Pb, and Zn in long-lived species, such as sea turtles [10, 70].

## Conclusions

The present study provides the first characterization of essential and non-essential element concentrations in the shell, pectoral muscle, liver, and kidney of *C. mydas* from the southern coast of Pernambuco, northeastern Brazil. The results demonstrate that this population is exposed to multiple environmental contaminants, with tissue-specific bioaccumulation patterns, particularly the preferential accumulation of Cd in the kidneys, highlighting the influence of regional environmental conditions on metal exposure.

Although toxic concentration thresholds have not yet been established for sea turtles, the elevated levels of some potentially toxic elements indicate that contaminant exposure may represent a health concern for this population. Because the different tissues analyzed reflect exposure over distinct temporal scales, the multi-tissue approach adopted in this study provides a comprehensive assessment of environmental contamination and metal bioaccumulation in *C. mydas*.

These findings reinforce the value of *C. mydas* as a sentinel species for coastal pollution and provide baseline data for future biomonitoring programs in the South Atlantic. Further studies integrating contaminant concentrations with physiological, immunological, reproductive, and behavioral endpoints are needed to establish toxicological thresholds and improve ecological risk assessments for sea turtles.

## Supplementary Information

Below is the link to the electronic supplementary material.

Supplementary file1 (PDF 131 KB)

## Data Availability

The datasets generated during and/or analysed during the current study are available from the corresponding author on reasonable request.

## References

[1] Cortés-Gómez AA, Romero D, Girondot M (2017) The current situation of inorganic elements in marine turtles: a general review and meta-analysis. Environ Pollut 229:567–585. 10.1016/j.envpol.2017.06.077 28688307

[2] D’Ilio S, Mattei D, Blasi MF, Alimonti A, Bogialli S (2011) The occurrence of chemical elements and POPs in loggerhead turtles (*Caretta caretta*): an overview. Mar Pollut Bull 62(8):1606–1615. 10.1016/j.marpolbul.2011.05.022 21700299

[3] Sinaei M, Bolouki M (2017) Metals in blood and eggs of green sea turtles (*Chelonia mydas*) from nesting colonies of the Northern Coast of the Sea of Oman. Arch Environ Contam Toxicol 73:552–561. 10.1007/s00244-017-0421-x 28631031

[4] Shaw KR, Lynch JM, Balazs GH, Jones TT, Pawloskie J, Ricef MR, French AD, Liu J, Cobb GP, Klein DM (2021) Trace element concentrations in blood and scute tissues from wild and captive Hawaiian green sea turtles (*Chelonia mydas*). Environ Toxicol Chem 40(1):208–218. 10.1002/etc.4911 PMC8452040 33103806

[5] Smith CE, Finlayson K, Barraza A, Young EJ, Gilby BL, Merwe JP, Townsend KA (2025) Distinct population-wide differences in contaminants and blood parameters in foraging green sea turtles. Mar Pollut Bull 212:117541. 10.1016/j.marpolbul.2025.117541 39813878

[6] Green SL, Beale DJ, Finlayson K, Shackleton M, Silvester E, Stauber J, van de Merwe J, Holland A (2026) Beneath the waves: a systematic review of the threats of bioaccumulative chemical contaminants to seagrass and herbivorous megafauna. Mar Pollut Bull 222(3):118790. 10.1016/j.marpolbul.2025.118790 41109174

[7] Miller DD (2017) Minerals. In: Damodaran S, Parkin KL (eds) Fennema’s food chemistry, 5th edn. CRC Press, Boca Raton, pp 627–679

[8] Underwood EJ, Mertz W (1987) Introduction. In: Mertz W (ed) Trace elements in human and animal nutrition, 5th edn. Academic Press, San Diego, pp 1–19

[9] Garrett RG (2000) Natural source of metals of the environment. Hum Ecol Risk Assess 6(6):945–963. 10.1080/10807030091124383

[10] Grillitsch B, Schiesari L (2010) The ecotoxicology of metals in reptiles. In: Sparling DW, Linder G, Bishop CA, Krest S (eds) Ecotoxicology of amphibians and reptiles, 2nd edn. CRC Press, Boca Raton, pp 337–448. 10.1201/EBK1420064162

[11] Haynes D, Johnson JE (2000) Organochlorine, heavy metal and polyaromatic hydrocarbon pollutant concentrations in the Great Barrier Reef (Australia) environment: a review. Mar Pollut Bull 41(7–12):267–278. 10.1016/S0025-326X(00)00134-X

[12] Tanabe LK, Scott K, Dasari V, Berumen ML (2022) An assessment of heavy metals in green sea turtle (*Chelonia mydas*) hatchlings from Saudi Arabia’s largest rookery, Ras Baridi. PeerJ 10:e13928. 10.7717/peerj.13928 PMC9415434 36032942

[13] Mondragón ME, Luzardo OP, Henríquez-Hernández LA, Rodríguez-Hernández A, Zumbado M, Martínez JRR, Farias FG, Suzán G, Islas CG (2023) Trophic behavior of inorganic elements in nesting sea turtles (*Chelonia mydas*, *Eretmochelys imbricata*, and *Caretta caretta*) in Quintana Roo: Biomagnification and biodilution effect in blood and scute tissues. Mar Pollut Bull 187:114582. 10.1016/j.marpolbul.2023.114582 36634539

[14] Wilkinson A, Ariel E, Merwe J, Brodie J (2023) Green turtle (*Chelonia mydas*) blood and scute trace element concentrations in the Northern Great Barrier Reef. Environ Toxicol Chem 42(11):2375–2388. 10.1002/etc.5718 37477460

[15] Fraga NS, Martins AS, Faust DR, Da Silva CC, Bianchini A, Aguirre AA, Sakai H (2024) Distribution of copper in the Atlantic and Pacific Oceans using green turtles (*Chelonia mydas*) as a bioindicator. Environ Sci Pollut Res 31:31967–31977. 10.1007/s11356-024-33366-y 38642227

[16] Hylland K (2006) Biological effects in the management of chemicals in the marine environment. Mar Pollut Bull 53(10–12):614–619. 10.1016/j.marpolbul.2006.08.010 16997329

[17] Ley-Quiñónez C, Zavala-Norzagaray AA, Espinosa-Carreón TL, Peckham H, Marquez-Herrera C, Campos-Villegas L, Aguirre AA (2011) Baseline heavy metals and metalloid values in blood of loggerhead turtles (*Caretta caretta*) from Baja California Sur, Mexico. Mar Pollut Bull 62(9):1979–1983. 10.1016/j.marpolbul.2011.06.022 21788056

[18] Finlayson KA, Leusch FDL, van de Merwe JP (2016) The current state and future directions of marine turtle toxicology research. Environ Int 94:113–123. 10.1016/j.envint.2016.05.013 27236406

[19] Jerez S, Motas M, Cánovas RÁ, Talavera J, Almela RM, del Río AB (2010) Accumulation and tissue distribution of heavy metals and essential elements in loggerhead turtles (*Caretta caretta*) from Spanish Mediterranean coastline of Murcia. Chemosphere 78(3):256–264. 10.1016/j.chemosphere.2009.10.062 19959203

[20] Maffucci F, Caurant F, Bustamante P, Bentivegna F (2005) Trace element (Cd, Cu, Hg, Se, Zn) accumulation and tissue distribution in loggerhead turtles (*Caretta caretta*) from the Western Mediterranean Sea (southern Italy). Chemosphere 58(5):535–542. 10.1016/j.chemosphere.2004.09.032 15620746

[21] Jian L, Zhang T, Lin L, Xiong J, Shi H, Wang J (2022) Transfer and accumulation of trace elements in seawater, sediments, green turtle forage, and eggshells in the Xisha Islands, South China Sea. Environ Sci Pollut Res 29:50832–50844. 10.1007/s11356-022-19354-0 35239116

[22] James MC, Sherrill-Mix SA, Martin K, Myers RA (2006) Canadian waters provide critical foraging habitat for leatherback sea turtles. Biol Conserv 133(3):347–357. 10.1016/j.biocon.2006.06.012

[23] Aguirre AA, Balazs GH, Zimmerman B, Spraker TR (1994) Evaluation of Hawaiian green turtle (*Chelonia mydas*) for potential pathogens associated with fibropapillomas. J Wildl Dis 30(1):8–15. 10.7589/0090-3558-30.1.8 8151829

[24] Anan Y, Kunito T, Sakai H, Tanabe S (2002) Subcellular distribution of trace elements in the liver of sea turtles. Mar Pollut Bull 45(1–12):224–229. 10.1016/S0025-326X(02)00106-6 12398389

[25] Franzellitti S, Locatelli C, Gerosa G, Vallini C, Fabbri E (2004) Heavy metals in tissues of loggerhead turtles (*Caretta caretta*) from the northwestern Adriatic Sea. Comp Biochem Physiol C Toxicol Pharmacol 138(2):187–194. 10.1016/j.cca.2004.07.008 15450866

[26] García-Fernández AJ, Gómez-Ramírez P, Martínez-López E, Hernández-García A, María-Mojica P, Romero D, Jiménez P, Castillo JJ, Bellido JJ (2009) Heavy metals in tissues from loggerhead turtles (*Caretta caretta*) from the southwestern Mediterranean (Spain). Ecotoxicol Environ Saf 72(2):557–563. 10.1016/j.ecoenv.2008.05.003 18571722

[27] Gardner SC, Fitzgerald SL, Vargas BA, Rodríguez LM (2006) Heavy metal accumulation in four species of sea turtles from the Baja California peninsula, Mexico. Biometals 19:91–99. 10.1007/s10534-005-8660-0 16502335

[28] Keller JM (2013) Exposure to and effects of persistent organic pollutants. In: Wyneken J, Lohmann KJ, Musick JA (eds) The biology of sea turtles, volume III, 1st edn. CRC Press, Boca Raton, pp 285–328

[29] Ley-Quiñónez CP, Zavala-Norzagaray AA, Réndon-Maldonado JG, Espinosa-Carreón TL, Canizales-Román A, Escobedo-Urías DC, Leal-Acosta ML, Hart CE, Aguirre AA (2013) Selected heavy metals and selenium in the blood of black sea turtle (*Chelonia mydas agasiizzi*) from Sonora, Mexico. Bull Environ Contam Toxicol 91:645–651. 10.1007/s00128-013-1114-4 24072261

[30] Pugh RS, Becker PR (2001) Sea turtle contaminants: a review with annotated bibliography. US Department of Commerce, National Institute of Standards and Technology. https://www.govinfo.gov/content/pkg/GOVPUB-C13-2d213a689722d820687d3bf8a62c4ba5/pdf/GOVPUB-C13-2d213a689722d820687d3bf8a62c4ba5.pdf. Accessed 26 January 2026

[31] Sakai H, Saeki K, Ichihashi H, Suganuma H, Tanabe S, Tatsukawa R (2000) Species-specific distribution of heavy metals in tissues and organs of loggerhead turtle (*Caretta caretta*) and green turtle (*Chelonia mydas*) from Japanese coastal waters. Mar Pollut Bull 40(8):701–709. 10.1016/S0025-326X(00)00008-4

[32] Villa CA, Flint M, Bell I, Hof C, Limpus CJ, Gaus C (2017) Trace element reference intervals in the blood of healthy green sea turtles to evaluate exposure of coastal populations. Environ Pollut 220(B):1465–1476. 10.1016/j.envpol.2016.10.085 27825845

[33] Villa CA, Bell I, Hof CM, Limpus CJ, Gaus C (2019) Elucidating temporal trends in trace element exposure of green turtles (*Chelonia mydas*) using the toxicokinetic differences of blood and scute samples. Sci Total Environ 651(2):2450–2459. 10.1016/j.scitotenv.2018.10.092 30336435

[34] Rowberry NB, Barraza AD, Limpus CJ, Merwe JP, Finlayson KA (2026) Trace element accumulation in green turtles (*Chelonia mydas*) foraging in locations of differing chemical input. Reg Stud Mar Sci 95:104817. 10.1016/j.rsma.2026.104817

[35] Fraga NS, Martins AS, Bianchini A, Faust DR, Sakai H, Da Silva CC, Aguirre AA (2023) Pantropical distribution of zinc in green turtles (*Chelonia mydas*): marine vertebrates as sentiel species. Environ Sci Pollut Res 30(17):50509–50519. 10.1007/s11356-023-25771-6 36795213

[36] Márquez MR (1990) Sea turtles of the world. FAO, Rome

[37] Bjorndal KA, Bolten AB, Lagueux CJ, Jackson DR (1997) Dietary overlap in three sympatric congeneric freshwater turtles (*Pseudemys*) in Florida. Chelonian Conserv Biol 2(3):430–433

[38] Bolten AB (2003) Variation in sea turtle life history patterns: neritic vs. oceanic development stages. In: Lutz PL, Musick JA, Wyneken J (eds) The biology of sea turtles, vol 2, 1st edn. CRC Press, Washington, pp 243–258

[39] Santos RG, Martins AS, Batista MB, Horta PA (2015) Regional and local factors determining green turtle *Chelonia mydas* foraging relationships with the environment. Mar Ecol Prog Ser 529:265–277. 10.3354/meps11276

[40] Zhang T, Lin L, Li D, Jian L, Li R, Wang J, Shi H (2022) Assessment of trace element contamination in the historical nesting grounds of green sea turtle (*Chelonia mydas*) in Hainan Island, China. Environ Sci Pollut Res 29:76394–76403. 10.1007/s11356-022-21057-5 35668262

[41] Talavera-Saenz A, Gardner SC, Rodriquez RR, Vargas BA (2007) Metal profiles used as environmental markers of green turtle (*Chelonia mydas*) foraging resources. Sci Total Environ 373(1):94–102. 10.1016/j.scitotenv.2006.10.012 17188334

[42] Adnyana W, Ladds PW, Blair D (1997) Observations of fibropapillomatosis in green turtles (*Chelonia mydas*) in Indonesia. Aust Vet J 75(10):737–742. 10.1111/j.1751-0813.1997.tb12258.x 9406633

[43] Van Houtan KS, Hargrove SK, Balazs GH (2010) Land use, macroalgae, and a tumor-forming disease in marine turtles. PLoS ONE 5(9):e12900. 10.1371/journal.pone.0012900 PMC2947502 20927370

[44] Foley AM, Schroeder BA, Redlow AE, Fick-Child KJ, Teas WG (2005) Fibropapillomatosis in stranded green turtles (*Chelonia mydas*) from the eastern United States (1980–98): trends and associations with environmental factors. J Wildl Dis 41(1):29–41. 10.7589/0090-3558-41.1.29 15827208

[45] Day RD, Segars AL, Arendt MD, Lee AM, Peden-Adams MM (2007) Relationship of blood mercury levels to health parameters in the loggerhead sea turtle (*Caretta caretta*). Environ Health Perspect 115(10):1421–1428. 10.1289/ehp.9918 PMC2022655 17938730

[46] Jones K, Ariel E, Burgess G, Read M (2016) A review of fibropapillomatosis in green turtles (*Chelonia mydas*). Vet J 212:48–57. 10.1016/j.tvjl.2015.10.041 27256025

[47] da Silva CC, Klein RD, Barcarolli IF, Bianchini A (2016) Metal contamination as a possible etiology of fibropapillomatosis in juvenile female green sea turtles *Chelonia mydas* sea turtles from the southern Atlantic Ocean. Aquat Toxicol 170:42–51. 10.1016/j.aquatox.2015.11.007 26615366

[48] Tagliolatto A, Guimaraes S, Lobo-Hajdu G, Monteiro-Neto C (2016) Characterization of fibropapillomatosis in green turtles *Chelonia mydas* (Cheloniidae) captured in a foraging area in southeastern Brazil. Dis Aquat Organ 121(3):233–240. 10.3354/dao03020 27786161

[49] Manes C, Pinton D, Canestrelli A, Capua I (2022) Occurrence of fibropapillomatosis in green turtles (*Chelonia mydas*) in relation to environmental changes in coastal ecosystems in Texas and Florida: a retrospective study. Animals (Basel) 12:1236. 10.3390/ani12101236 PMC9137486 35625082

[50] Roost T, Schies J, Girondot M, Robin J, Lelong P, Martin J, Siegwalt F, Jeantet L, Giraudeau M, Le Loch G, Bejarano M, Bonola M, Benhalilou A, Murgale C, Andreani L, Jacaria F, Campistron G, Lathiere A, Martial F, Hielard G, Arque A, Regis S, Lecerf N, Frouin C, Lefebvre F, Aubert N, Flora F, Pimentel E, Lafolle R, Thobor F, Arthus M, Etienne D, Lecerf N, Allenou J, Desigaux F, Larcher E, Larcher C, Lo Curto A, Befort J, Maceno-Panevel M, Lepori M, Chevallier P, Chevallier T, Meslier S, Landreau A, Habold C, Le Maho Y, Chevallier D (2022) Fibropapillomatosis prevalence and distribution in immature green turtles (*Chelonia mydas*) in Martinique Island (Lesser Antilles). EcoHealth 19:190–202. 10.1007/s10393-022-01601-y 35665871

[51] Tenería FAM, Labrada-Martagón V, Herrera-Pavón RL, Work TM, González-Ballesteros E, Negrete-Philippe AC, Maldonado-Saldaña G (2022) Fibropapillomatosis dynamics in green sea turtles *Chelonia mydas* over 15 years of monitoring in Akumal Bay, Quintana Roo, Mexico. Dis Aquat Organ 149:133–143. 10.3354/dao03669 35735233

[52] Pérez YAA, Lima SR, Souza GM, Gião T, Bispo FJS, Reis AMF, Leite JS, Silva KVGC (2023) Evaluation of biometry and blood concentration of heavy metals in free-living *Chelonia mydas* with and without fibropapillomatosis in southern Brazil. Mar Pollut Bull 190:114879. 10.1016/j.marpolbul.2023.114879 37004471

[53] Vanstreels RET, Durant A, Santos AP, Santos RG, Sarmiento AMS, Rossi S, Setim FE, Gattamorta MA, Matushima ER, Mayorga LFSP, Uhart MM (2023) Exploring the relationship between environmental drivers and the manifestation of fibropapillomatosis in green turtles (*Chelonia mydas*) in eastern Brazil. PLoS ONE 18(8):e0290312. 10.1371/journal.pone.0290312 PMC10449228 37616208

[54] Wibbels T, Owens DW, Limpus CJ (2000) Sexing juvenile sea turtles: is there an accurate and practical method? Chelonian Conserv Biol 3(4):756–761

[55] Wyneken J, Epperly SP, Crowder LB, Vaughan J, Esper KB (2007) Determining sex in posthatchling loggerhead sea turtles using multiple gonadal and accessory duct characteristics. Herpetologica 63(1):19–30. 10.1655/0018-0831(2007)63[19:DSIPLS]2.0.CO;2

[56] Bolten AB (1999) Techniques for measuring sea turtles. In: Eckert KL, Bjorndal KA, Abreu-Grobois FA, Donnelly M (eds) Research and management techniques for the conservation of sea turtles, 1st edn. IUCN/SSC Marine Turtle Specialist Group Publication No. 4., Washington, pp 110–114

[57] da Silva IJS, Lavorante AF, Paim APS, da Silva MJ (2020) Microwave-assisted digestion employing diluted nitric acid for mineral determination in rice by ICP OES. Food Chem 319:126435. 10.1016/j.foodchem.2020.126435 32155538

[58] da Silva IJS, da Silva MM, da Silva RGMF, De França EJ, da Silva MJ, Kato MT (2025) Microwave-assisted digestion for multi-elemental determination in beans, basil, and mint by ICP OES and flame photometry: an eco-friendly alternative. Food Chem 481:143970. 10.1016/j.foodchem.2025.143970 40154058

[59] Analytical Methods Committee (1987) Recommendations for the definition, estimation and use of the detection limit. Anal 112(2):199–204. 10.1039/an9871200199

[60] Eurachem (2014). The fitness for purpose of analytical methods: a laboratory guide to method validation and related topics, Second edn. https://www.eurachem.org/images/stories/Guides/pdf/MV_guide_2nd_ed_EN.pdf. Accessed 14 July 2026

[61] Grossman A, Mendonça P, da Costa MR, Bellini C (2007) Morphometrics of the green turtle at the Atol das Rocas Marine Biological Reserve, Brazil. Mar Turtle Newsl 118:12–13

[62] Barin JS, Bizzi CA, Flores EMM, Nóbrega JA, Krug FJ (2019) Decomposição de materiais orgânicos por via úmida. In: Krug FJ, Rocha FRP (eds) Métodos de preparo de amostras para análise elementar, 2nd edn. EditSBQ, São Paulo, pp 263–298

[63] da Fonseca CF, da Silva IJS, Rodrigues MS, Silva BHS, Soares PC, de Moura GJB, de Oliveira JB (2023) Evaluation of metal exposure through the composition of essential and toxic micro-minerals in freshwater turtles (*Phrynops geoffroanus*) from a Brazilian river. Environ Sci Pollut Res 30:54871–54884. 10.1007/s11356-023-26127-w 36881237

[64] Yang SGNS, da Silva IJS, Souza DS, Fonseca CF, Santiago ACS, Soares PC, Oliveira JB (2021) Multi-elemental exposure assessment through concentration in hair of free-ranging capybaras (*Hydrochoerus hydrochaeris*, Linnaeus, 1766) in the Atlantic Forest remants. Northeast of Brazil Chemosphere 262:127800. 10.1016/j.chemosphere.2020.127800 32750592

[65] Wiltsche H, Winkler M, Tirk P (2015) Matrix effects of carbon and bromine in carbon and bromine in inductively coupled plasma optical emission spectrometry. J Anal At Spectrom 30:2223–2234. 10.1039/c5ja00237k

[66] Komoroske LM, Lewison RL, Seminoff JA, Deustchman DD, Deheyn DD (2012) Trace metals in an urbanized estuarine sea turtle food web in San Diego Bay, CA. Sci Total Environ 417–418:108–116. 10.1016/j.scitotenv.2011.12.018 22261404

[67] Andreani G, Santoro M, Cottignoli S, Fabbri M, Carpenè E, Isani G (2008) Metal distribution and metallothionein in loggerhead (*Caretta caretta*) and green (*Chelonia mydas*) sea turtles. Sci Total Environ 390(1):287–294. 10.1016/j.scitotenv.2007.09.014 18022677

[68] Bezerra MF, Lacerda LD, Costa BGB, Lima EHSM (2012) Mercury in the sea turtle *Chelonia mydas* (Linnaeus, 1958) from Ceará coast, NE Brazil. An Acad Bras Ciênc 84(1):123–128. 10.1590/S0001-37652012000100012 22441601

[69] Ikonomopoulou MP, Olszowy H, Francis R, Ibrahim K, Whittier J (2013) Accumulation of trace metals in the embryos and hatchlings of *Chelonia mydas* from Peninsular Malaysia incubated at different temperatures. Sci Total Environ 450–451:301–306. 10.1016/j.scitotenv.2013.02.031 23500829

[70] Silva CC, Varela AS Jr, Barcarolli IF, Bianchini A (2014) Concentrations and distributions of metals in tissues of stranded green sea turtles (*Chelonia mydas*) from the southern Atlantic coast of Brazil. Sci Total Environ 466–467:109–118. 10.1016/j.scitotenv.2013.06.094 23895781

[71] Camacho M, Boada LD, Orós J, López P, Zumbado M, Almeida-González M, Luzardo OP (2014) Monitoring organic and inorganic pollutants in juvenile live sea turtles: results from a study of *Chelonia mydas* and *Eretmochelys imbricata* in Cape Verde. Sci Total Environ 481:303–310. 10.1016/j.scitotenv.2014.02.051 24602915

[72] Yipel M, Tekeli İO, İşler CT, Altuğ ME (2017) Heavy metal distribution in blood, liver and kidneys of Loggerhead (*Caretta caretta*) and Green (*Chelonia mydas*) sea turtles from the Northeast Mediterranean Sea. Mar Pollut Bull 125(1–2):487–491. 10.1016/j.marpolbul.2017.08.011 28802660

[73] Souza NLN, Carneiro MTWD, Pimentel EF, Frossard A, Freire JB, Endringer DC, Ferreira Júnior PD (2018) Trace elements influence the hatching success and emergence of *Caretta caretta* and *Chelonia mydas*. J Trace Elem Med Biol 50:117–122. 10.1016/j.jtemb.2018.06.007 30262268

[74] Macêdo GR, Tarantino TB, Barbosa IS, Pires TT, Rostan G, Goldberg DW, Pinto LFB, Korn MGA, Franke CR (2015) Trace elements distribution in hawksbill turtle (*Eretmochelys imbricata*) and green turtle (*Chelonia mydas*) tissues on the northern coast of Bahia. Brazil Mar Pollut Bull 94(1–2):284–289. 10.1016/j.marpolbul.2015.02.033 25796544

[75] Barraza AD, Komoroske LM, Allen C, Eguchi T, Gossett R, Holland E, Lawson DD, LeRoux RA, Long A, Seminoff JA, Lowe CG (2019) Trace metals in green sea turtles (*Chelonia mydas*) inhabiting two southern California coastal estuaries. Chemosphere 223:342–350. 10.1016/j.chemosphere.2019.01.107 PMC6620110 30784740

[76] Sinaei M, Zare R, Matin MT, Ghasemzadeh J (2021) Marine debris and trace metal (Cu, Cd, Pb, and Zn) pollution in the stranded green sea turtles (*Chelonia mydas*). Arch Environ Contam Toxicol 80:634–644. 10.1007/s00244-021-00829-z 33751194

[77] Farias DSD, Rossi S, Bomfim AC, Fragoso ABL, Santos-Neto EB, Silva FJL, Lailson-Brito J, Navoni JA, Gavilan SA, Amaral VS (2022) Bioaccumulation of total mercury, copper, cadmium, silver, and selenium in green turtles (*Chelonia mydas*) stranded along the Potiguar Basin, northeastern Brazil. Chemosphere 299:134331. 10.1016/j.chemosphere.2022.134331 35339524

[78] Çelik S, Beton D, Çiçek BA, Snape RTE, Baskale E (2023) Metal accumulation in juvenile and sub-adult loggerhead and green turtles in northern Cyprus. Environ Pollut 316:120482. 10.1016/j.envpol.2022.120482 36279995

[79] Tostes ECL, Viana LMS, Agostinho KFF, Almeida CMS, Carvalho CEV (2023) Trace element bioaccumulation in the hepatic tissue of juvenile green turtles (*Chelonia mydas*) stranded along the Campos and Espírito Santo basins, southeastern Brazil. Mar Pollut Bull 195:115447. 10.1016/j.marpolbul.2023.115447 37716129

[80] Beauvieux A, Bourjea J, Fromentin J, Jean C, Ciccione S, Ballorain K, Romero D, Dbouk Z, Hirschler A, Bertile F, Schull Q (2024) Tracing troubles: unveiling the hidden impact of inorganic contamination on juvenile green sea turtle. Mar Pollut Bull 208:117048. 10.1016/j.marpolbul.2024.117048 39368147

[81] Morão IFC, Simões T, Casado RB, Vieira S, Ferreira-Airaud B, Caliani I, Di Noi A, Casini S, Fossi MC, Lemos MFL, Novais SC (2024) Metal accumulation in female green sea turtles (*Chelonia mydas*) from Eastern Atlantic affects their egg quality with potential implications for embryonic development. Sci Total Environ 931:172710. 10.1016/j.scitotenv.2024.172710 38670375

[82] Chan Y, Robinson NJ, Dourdeville K, Haas HL, Nielsen J, Paladino FV, Prescott R, Patel SH (2025) Heavy metal concentrations suggest pollution risk varies between sea turtle species in the northwest Atlantic Ocean. Chemosphere 373:144190. 10.1016/j.chemosphere.2025.144190 39908845

[83] Li X, Jian L, Fu M, Zhang H, Zhang Y, Guo R, Wang J (2025) Study on the distribution characteristics and toxic risks of trace elements in the eggs of green turtles (*Chelonia mydas*) nesting on the Xisha Islands. Environ Res 285:122418. 10.1016/j.envres.2025.122418 40706835

[84] Silverman J, Levin O, Levy Y, Rybak O, Segal Y, Shachnai A, Asfur M (2025) Total mercury levels in loggerhead and green sea turtle hatched eggshells from nests along the southeastern Mediterranean coast. Chemosphere 390:144705. 10.1016/j.chemosphere.2025.144705 41075630

[85] Ross DA, Guzmán HC, Van Hinsberg VJ, Potvin C (2016) Metal contents of marine turtle eggs (*Chelonia mydas*; *Lepidochelys olivacea*) from the tropical eastern pacific and the implications for human health. J Environ Sci Health B 51(10):675–687. 10.1080/03601234.2016.1191888 27323656

[86] Shi X, Huang D, Wang C, Xiong J, Jian L, Wang J (2026) Variations in the distribution of trace elements in coral sand sediments at the green turtle nesting ground in the Xisha Islands, South China Sea. Mar Pollut Bull 223:119046. 10.1016/j.marpolbul.2025.119046 41317643

[87] Lacerda LD, Santos JA, Campos RC, Gonçalves RA, Salles R (2007) Total-Hg and organic-Hg in *Cephalopholis fulva* (Linnaeus, 1758) from inshore and offshore waters of NE Brazil. Braz J Biol 67(3):493–498. 10.1590/S1519-69842007000300014 18094832

[88] Barbieri E (2009) Concentration of heavy metals in tissues of green turtles (*Chelonia mydas*) sampled in the Cananéia estuary, Brazil. Braz J Oceanogr 57(3):243–248. 10.1590/S1679-87592009000300007

[89] Bellini C, Santos AJB, Grossman A, Marcovaldi MA, Barata PCR (2013) Green turtle (*Chelonia mydas*) nesting on Atol das Rocas, north-eastern Brazil, 1990–2008. J Mar Biol Assoc U K 93(4):1117–1132. 10.1017/S002531541200046X

[90] Agostinho KFF, Lacerda D, Tostes ECL, Baldassin P, Di Beneditto APM, Carvalho CEV (2020) Trace elements in green turtles (*Chelonia mydas*) from Rocas Atoll, NE Brazil: baseline reference from a pristine nesting site. Mar Pollut Bull 157:111271. 10.1016/j.marpolbul.2020.111271 32469744

[91] Agostinho KFF, Pestana IA, Carvalho CEV, Di Beneditto APM (2021) Trace elements and stable isotopes in egg yolk of green turtles on Rocas Atoll, Brazil. Mar Pollut Bull 162:111821. 10.1016/j.marpolbul.2020.111821 33162056

[92] Verzele P, Rodriguez CB, Bezerra MF, Lacerda LD (2024) Regional differences in Hg contents in the liver and muscle of the green turtle (*Chelonia mydas*) from three foraging grounds in Northeast Brazil. Environ Sci Pollut Res 31:3917–3925. 10.1007/s11356-023-31407-6 38095795

[93] Brito GB, Souza TL, Costa FN, Moura CW, Korn MG (2016) Baseline trace elements in the seagrass Halodule wrightii Aschers (*Cymodoceaceae*) from Todos os Santos Bay, Bahia. Brazil Mar Pollut Bull 104(1–2):335–342. 10.1016/j.marpolbul.2016.01.044 26874749

[94] Rodriguez CAB, Bezerra MF, Rezende CED, Bastos WR, Lacerda LD (2019) Mercury and methylmercury in carapace of the marine turtle *Caretta caretta*, in northeastern Brazil and its potential for environmental monitoring. An Acad Bras Cienc 91(2):e20180672. 10.1590/0001-3765201920180672 31291384

[95] Rodriguez CAB, De Lacerda LD, Bezerra MF, Moura VL, Rezende CE, Bastos WR (2020) Influence of size on total mercury (THg), methyl mercury (MeHg), and stable isotopes of N and C in green turtles (*Chelonia mydas*) from NE Brazil. Environ Sci Pollut Res 27:20527–20537. 10.1007/s11356-020-08623-5 32242319

[96] Bruno DA, Willmer IQ, Pereira LHSS, Rafael CC, Rocha RCC, Saint’Pierre TD, Baldassin O, Scarelli ACS, Tadeu AD, Correia FV, Saggioro EM, Lemos LS, Siciliano S, Hauser-Davis RA (2021) Metal and metalloid contamination in green sea turtles (*Chelonia mydas*) found stranded in Southeastern Brazil. Front Mar Sci 8:608253. 10.3389/fmars.2021.608253

[97] Miguel C, Costa PG, Bianchini A, Luzardo OLP, Vianna MRM, Santos MRD (2022) Health condition of *Chelonia mydas* from a foraging area affected by the tailings of a collapsed dam in Southeast Brazil. Sci Total Environ 821:153353. 10.1016/j.scitotenv.2022.153353 35085636

[98] Barrios-Rodríguez CA, de Lacerda LD, Fernandes-Bezerra MA (2023) Pilot study of mercury distribution in the carapace of four species of sea turtles from northeastern Brazil. Bull Environ Contam Toxicol 110:99. 10.1007/s00128-023-03745-1 37243788

[99] Nunes VL, Cutrim CHG, Koch EBA, Araújo VA (2023) Anthropogenic threats associated with mortality and biomass of stranded sea turtles on the northern coast of São Paulo State. Brazil Int J Environ Stud 80(6):1–15. 10.1080/00207233.2023.2216609

[100] Lam JCW, Tanabe S, Chan SKF, Yuen EKW, Lam MHW, Lam PKS (2004) Trace element residues in tissues of green turtles (*Chelonia mydas*) from South China waters. Mar Pollut Bull 48(1–2):174–182. 10.1016/j.marpolbul.2003.09.003 14725889

[101] Storelli MM, Barone G, Storelli A, Marcotrigiano GO (2008) Total and subcellular distribution of trace elements (Cd, Cu and Zn) in the liver and kidney of green turtles (*Chelonia mydas*) from the Mediterranean Sea. Chemosphere 70:908–913. 10.1016/j.chemosphere.2007.06.069 17707456

[102] Anan Y, Kunito T, Watanabe I, Sakai H, Tanabe S (2001) Trace element accumulation in hawksbill turtle (*Eretmochelys imbricata*) and green turtle (*Chelonia mydas*) from Yaeyama Islands. Japan Environ Toxicol Chem 20(12):2802–2814. 10.1002/etc.5620201220 11764164

[103] Carpenè E, Serra R, Isani G (1995) Effects of heavy metals on algae. In: Cheremisinoff PN (ed) Encyclopedia of environmental control technology, 1st edn. Gulf Publishing Company, Houston, pp 707–759

[104] Roberts DA, Johnston EL, Poore AGB (2008) Biomonitors and the assessment of ecological impacts: distribution of herbivorous epifauna in contaminated macroalgal beds. Environ Pollut 156(2):489–503. 10.1016/j.envpol.2008.01.012 18281133

[105] Caurant F, Bustamante P, Bordes M, Miramand P (1999) Bioaccumulation of cadmium, copper and zinc in some tissues of the three species of marine turtles stranded along the French Atlantic coasts. Mar Pollut Bull 38(12):1085–1091. 10.1016/S0025-326X(99)00109-5

[106] Sakai H, Saeki K, Ichihashi H, Kamezaki N, Tanabe S, Tatsukawa R (2000) Growth-related changes in heavy metal accumulation in green turtle (*Chelonia mydas*) from Yaeyama Islands, Okinawa, Japan. Arch Environ Contam Toxicol 39:378–385. 10.1007/s002440010118 10948289

[107] Meyers-Schöne L, Walton BT (1994) Turtles as monitors of chemical contaminants in the environment. In: Ware GW (ed) Reviews of environmental contamination and toxicology, vol 135, 1st edn. Springer, New York, pp 93–153. 10.1007/978-1-4612-2634-5_3

[108] Guirlet E, Das K (2012) Cadmium toxicokinetics and bioaccumulation in turtles: trophic exposure of *Trachemys scripta elegans*. Ecotoxicology 21:18–26. 10.1007/s10646-011-0758-2 21814846

[109] Yaghmour F, Al Bousi M, Whittington-Jones B, Pereira J, García-Nuñez S, Budd J (2018) Marine debris ingestion of green sea turtles, *Chelonia mydas*, (Linnaeus, 1758) from the eastern coast of the United Arab Emirates. Mar Pollut Bull 135:55–61. 10.1016/j.marpolbul.2018.07.013 30301072

[110] Nicolau L, Monteiro SS, Pereira AT, Marçalo A, Ferreira M, Torres J, Vingada J, Eira C (2017) Trace elements in loggerhead turtles (*Caretta caretta*) stranded in mainland Portugal: bioaccumulation and tissue distribution. Chemosphere 179:120–126. 10.1016/j.chemosphere.2017.03.108 28364647

[111] Abdallah MAM (2023) Bioaccumulation and biomagnifications of toxic metals in tissues of loggerhead turtles (*Caretta caretta*) from the Mediterranean Sea coast, Egypt. Sci Rep 13:7995. 10.1038/s41598-023-33972-9 PMC10192338 37198257

[112] Nordberg G (1972) Cadmium metabolisms and toxicity. Environ Physiol Biochem 2:7–36

[113] Aguirre AA, Balasz GH, Zimmerman B, Galy FD (1994) Organic contaminants and trace metals in the tissues of green turtles (*Chelonia mydas*) afflicted with fibropapillomas in the Hawaiian islands. Mar Pollut Bull 28(2):109–114. 10.1016/0025-326X(94)90547-9

[114] Gordon AN, Pople AR, Ng J (1998) Trace metal concentrations in livers and kidneys of sea turtles from south-eastern Queensland, Australia. Mar Freshw Res 49(5):409–414. 10.1071/MF97266

[115] Godley BJ, Thompson DR, Furness RW (1999) Do heavy metal concentrations pose threat to marine turtle from Mediterranean Sea? Mar Pollut Bull 38(6):497–502. 10.1016/S0025-326X(98)00184-2

[116] Simsek I, Kuzukiran O, Filazi I, Boztepe UG, Ozen D, Kara E, Filazi A (2025) Assessment of metal distribution patterns in loggerhead sea turtles: a comparative analysis based on sex and tissue type. Mar Pollut Bull 219:118339. 10.1016/j.marpolbul.2025.118339 40561618

[117] Faust DR, Hooper MJ, Cobb GP, Barnes M, Ertolacci DSS, Smith PN (2014) Inorganic elements in green sea turtles (*Chelonia mydas*): relationships among external and internal tissues. Environ Toxicol Chem 33:2020–2027. 10.1002/etc.2650 24889685

[118] Storelli MM, Marcotrigiano GO (2003) Heavy metal residues in tissues of marine turtles. Mar Pollut Bull 46(4):397–400. 10.1016/S0025-326X(02)00230-8 12705911

[119] Fraga NS, Martins AS, Faust DR, Sakai H, Bianchini A, Da Silva CC, Aguirre AA (2018) Cadmium in tissues of green turtles (*Chelonia mydas*): a global perspective for marine biota. Sci Total Environ 637:389–397. 10.1016/j.scitotenv.2018.04.317 29753227

[120] Suttle NF (2010) Mineral nutrition of livestock. CABI Publishing, Oxforshire

[121] Torrent A, González-Díaz OM, Monagas P, Orós J (2004) Tissue distribution of metals in loggerhead turtles (*Caretta caretta*) stranded in the Canary Islands. Spain Mar Pollut Bull 49(9–10):854–860. 10.1016/j.marpolbul.2004.08.022 15530530

[122] Homer BL, Foley A, Fick KJ, Lores MC, Redlow AE, Jacobson ER (2000) Lesions, pathogens and toxins identified in 13 stranded marine turtles in Florida. In: Abreu-Grobois FA, Briseño-Dueñas R, Márquez-Millán R, Sarti-Matínez L (eds) Proceedings of the Eighteenth International Sea Turtle Symposium, 1st edn. U.S. Department of Commerce, NOAA Technical Memorandum NMFS-SEFSC-436, Miami, pp 117–118

[123] Rodrigues MS, Cocentino ALM, Fonseca CF, Moura GJB, Fernandes MLB, Melo TMB, Queiroz EP, Silva AC (2019) Contribution to the study of the green turtle (*Chelonia mydas*) diet in the South Atlantic, Northeast Brazil. Intern J of Dev Res 9(9):29891–29897

[124] Brito GB, de Souza TL, Bressy FC, Moura CWN, Korn MGA (2012) Levels and spatial distribution of trace elements in macroalgae species from the Todos os Santos Bay, Bahia, Brazil. Mar Pollut Bull 64(10):2238–2244. 10.1016/j.marpolbul.2012.06.022 22795614

[125] Burton GA, Johnston EL (2010) Assessing contaminated sediments in the context of multiple stressors. Environ Toxicol Chem 29(12):2625–2643. 10.1002/etc.332 20931605

[126] Barbieri E (2009) Effects of zinc and cadmium on oxygen consumption and ammonium excretion in Barbieri pink shrimp (*Farfantepenaeus paulensis*, Pérez-Farfante, 1967, Crustacea). Ecotoxicology 18:312–318. 10.1007/s10646-008-0285-y 19031115

[127] Labrada-Martagón V, Rodríguez PAT, Méndez-Rodríguez LC, Zenteno-Savín T (2011) Oxidative stress indicators and chemical contaminants in East Pacific green turtles (*Chelonia mydas*) inhabiting two foraging coastal lagoons in the Baja California peninsula. Comp Biochem Physiol C Toxicol Pharmacol 154(2):65–75. 10.1016/j.cbpc.2011.02.006 21377544

[128] Pyle GG, Couture P (2012) Homeostasis and toxicology of essential metals. In: Wood CM, Farrell AP, Brauner CJ (eds) Fish physiology, 1st edn. Academic Press, Amsterdam, pp 253–289

[129] Goyer RA, Clarkson TW (2001) Toxic effects of metals. In: Klaasen CD (ed) Casarett and Doull’s toxicology: the basic science of poisons, 6th edn. McGraw-Hill, New York, pp 861–867

[130] Baker AJM, McGrath SP, Reeves RD, Smith JAC (2000) Metal hyperaccumulator plants: a review of the ecology and physiology of a biochemical resource for phytoremediation of metal-polluted soils. In: Terry N, Bañuelos G (eds) Phytoremediation of contaminated soil and water, 1st edn. CRC Press, Boca Raton, pp 85–107

[131] Sharma RP (2007) Immunotoxicity. In: Gupta RC (ed) Veterinary toxicology: basic and clinical principles, 1st edn. Academic Press, New York, pp 289–301

[132] Gissi F, Stauber JL, Binet MT, Golding LA, Adams MS, Schlekat CE, Garman ER, Jolley DF (2016) A review of nickel toxicity to marine and estuarine tropical biota with particular reference to the South East Asian and Melanesian region. Environ Pollut 218:1308–1323. 10.1016/j.envpol.2016.08.089 27622840

[133] Karthikeyan P, Marigoudar SR, Nagarjuna A, Sharma KV (2019) Toxicity assessment of cobalt and selenium on marine diatoms and copepods. Environ Chem Ecotox 1:36–42. 10.1016/j.enceco.2019.06.001

[134] Gaus C, Villa CA, Dogruer G, Heffernan A, Vijayasarathy S, Lin CY, Flint M, Hof CM, Bell I (2019) Evaluating internal exposure of sea turtles as model species for identifying regional chemical threats in nearshore habitats of the Great Barrier Reef. Sci Total Environ 658:732–743. 10.1016/j.scitotenv.2018.10.257 30583168

[135] Eisler R (2000) Handbook of chemical risk assessment. Lewis Publishers, Boca Raton

[136] Niemuth JN, Sanders CW, Mooney CB, Olfenbuttel C, DePerno CS, Stoskopf MK (2014) Nephrolithiasis in free-ranging North American river otter (*Lontra canadensis*) in North Carolina, USA. J Zoo Wildl Med 45(1):110–117. 10.1638/2013-0135R2.1 24712169

[137] Agência Nacional de Vigilância Sanitária – ANVISA (2022). Resolução da Diretoria Colegiada - RDC Nº 722, de 1° de julho de 2022. https://in.gov.br/en/web/dou/-/resolucao-rdc-n-722-de-1-de-julho-de-2022-413365215. Accessed 02 April 2026

[138] Agência Nacional de Vigilância Sanitária – ANVISA (2022). Instrução Normativa - IN Nº 160, de 1° de julho de 2022. https://www.in.gov.br/en/web/dou/-/instrucao-normativa-in-n-160-de-1-de-julho-de-2022-413367081. Accessed 02 April 2026

[139] Jakimska A, Konieczka P, Skóra K, Namieśnik J (2011) Bioaccumulation of metals in tissues of marine animals, Part I: the role and impact of heavy metals on organisms. Pol J Environ Stud 20(5):1117–1125. https://www.pjoes.com/pdf-88659-22518. Accessed 18 Aug 2026

