# Supplementary material for: Assessment of Exposure to Essential and Non-Essential Elements through Concentrations in Tissues of Chelonia mydas (Green turtles) from the South Atlantic, Northeast Brazil
Source: Biol Trace Elem Res. 2026 Aug 19;204(11):8850–73. doi: 10.1007/s12011-026-05303-2 (PMC13714022; doi:10.1007/s12011-026-05303-2)

Assessment of exposure to essential and non-essential elements through concentrations in tissues of *Chelonia mydas* (green turtles) from the South Atlantic, Northeast Brazil

Journal: Biological Trace Element Research

Midiã S. Rodrigues[1,2], Iago J.S. da Silva[3], Cristina F. da Fonseca[4], Pierre C. Soares[5*], Múcio L.B. Fernandes[6], Geraldo J.B. de Moura[1,2,7*]

[1]Programa de Pós-Graduação em Ciência Animal Tropical (PGCAT), Universidade Federal Rural de Pernambuco (UFRPE), Rua Dom Manoel de Medeiros, s/n, 52171-9000, Dois Irmãos, Recife, Pernambuco, Brazil

[2]Laboratório de Estudos Herpetológicos e Paleherpertológicos (LEHP), Universidade Federal Rural de Pernambuco (UFRPE), Rua Dom Manoel de Medeiros, s/n, 52171-9000, Dois Irmãos, Recife, Pernambuco, Brazil

[3]Unidade Acadêmica de Belo Jardim (UABJ), Universidade Federal Rural de Pernambuco (UFRPE), Rodovia PE 166, km 5, Euno Andrade da Silva, 55156-580, Belo Jardim, Pernambuco, Brazil

[4]Laboratório de Parasitologia (LAPAR), Departamento de Biologia, Universidade Federal Rural de Pernambuco (UFRPE), Rua Dom Manoel de Medeiros, s/n, 52171-9000, Dois Irmãos, Recife, Pernambuco, Brazil

[5]Departamento de Medicina Veterinária, Universidade Federal Rural de Pernambuco (UFRPE), Rua Dom Manoel de Medeiros, s/n, 52171-9000, Dois Irmãos, Recife, Pernambuco, Brazil

[6]Laboratório de Estudos Ambientais, Universidade de Pernambuco (UPE), Rua Amaro Maltês de Farias, 55800-000, Nazaré da Mata, Pernambuco, Brazil

[7]Programa de Pós-Graduação em Ecologia (PPGE), Universidade Federal Rural de Pernambuco (UFRPE), Rua Dom Manoel de Medeiros, s/n, 52171-9000, Dois Irmãos, Recife, Pernambuco, Brazil

*Corresponding authors:

Pierre C. Soares: pierre.soares@ufrpe.br; Geraldo J.B. de Moura: geraldo.jbmoura@ufrpe.br

Supplementary Material:

Plotting of PCA scores and loadings for kidney samples

Fig. S1 Scores plot of PCA for kidney samples specified by location of stranding: area 1 (blue boxes), area 2 (red dots), and area 3 (green triangles). PC - principal component

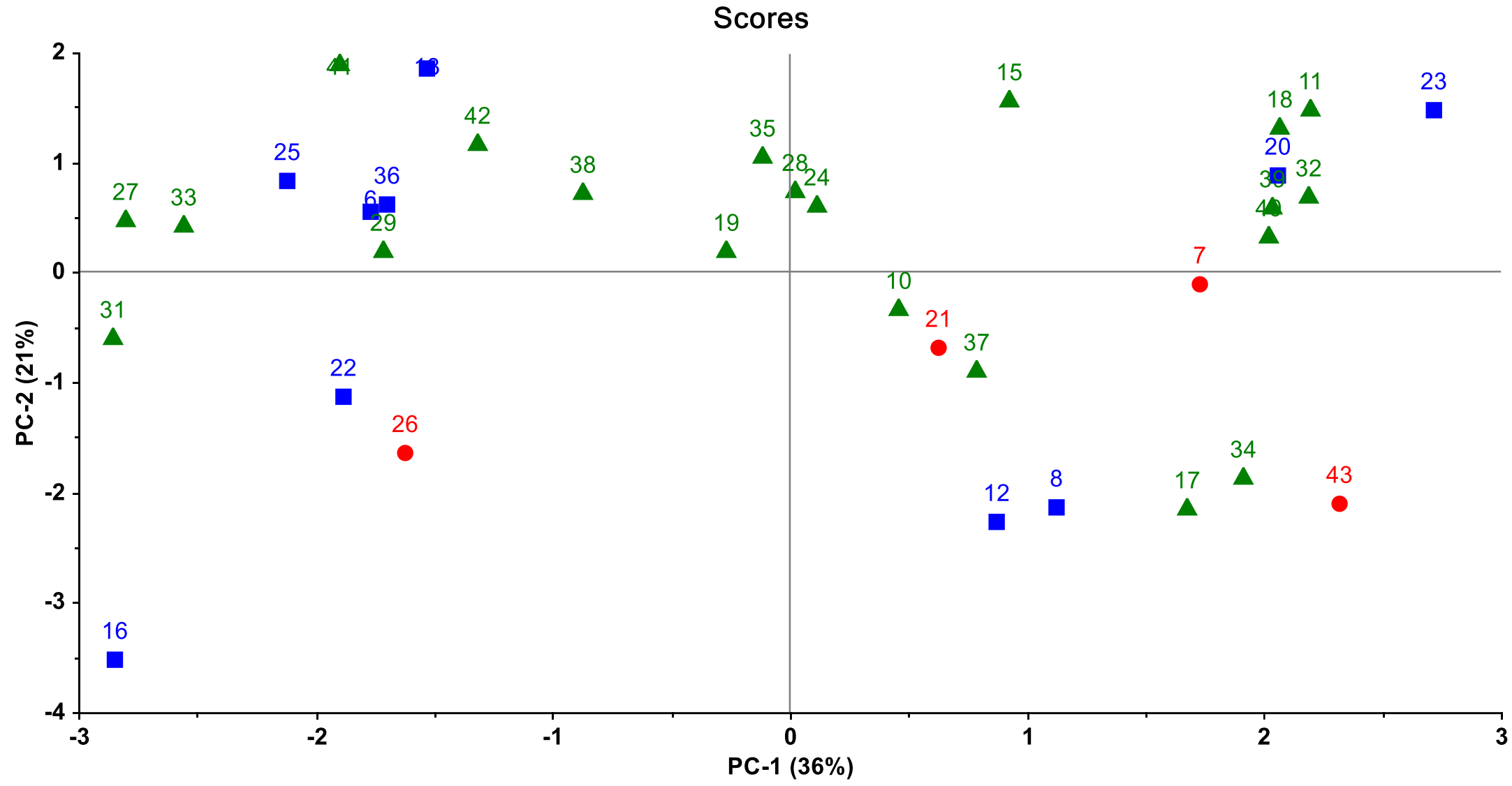

Fig. S2 Loadings plot of PCA for kidney samples

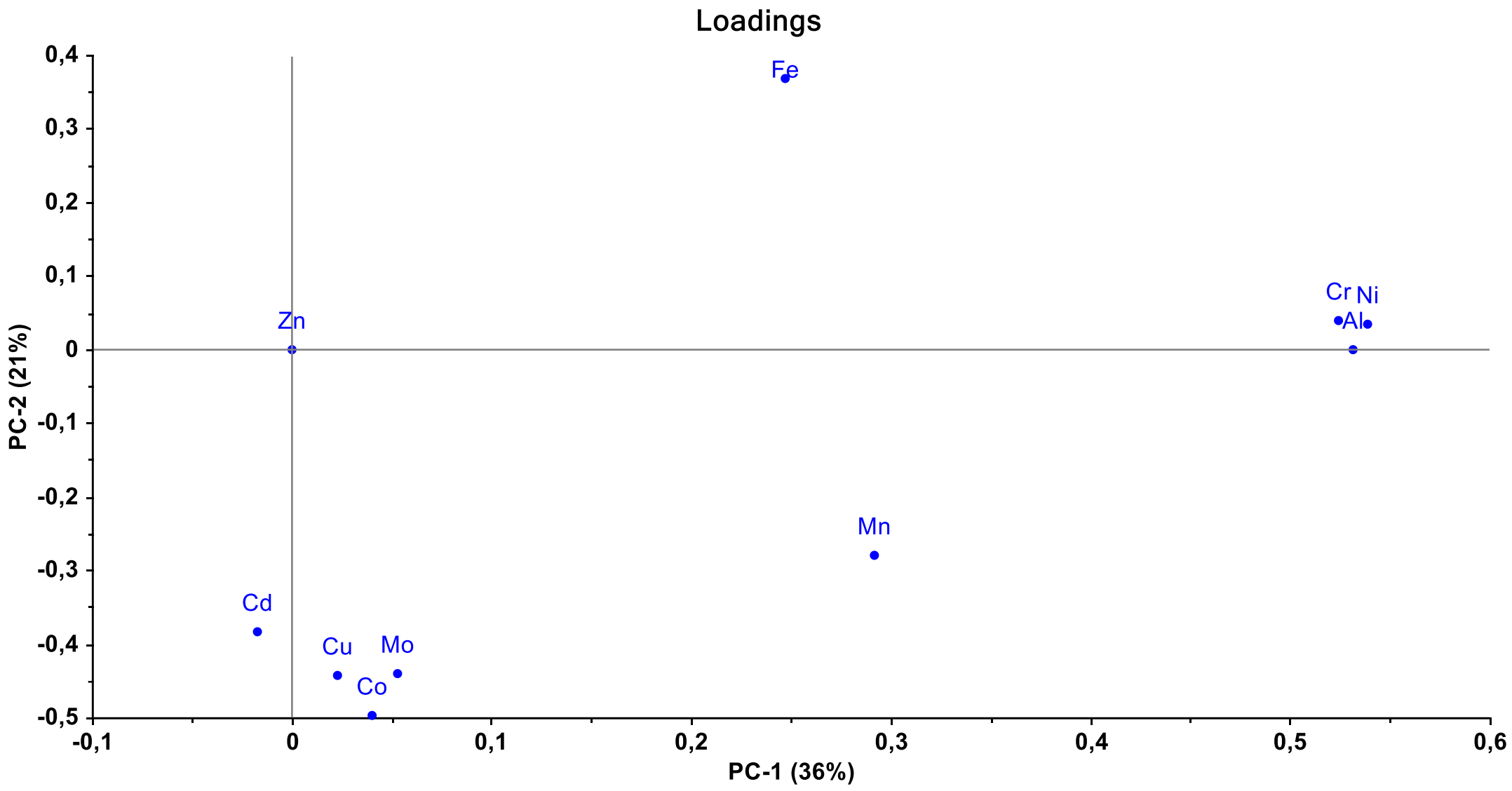

Supplement: Supplementary file 1 [file 12011_2026_5303_MOESM1_ESM.pdf]
